# Interventions to promote health literacy among working-age populations experiencing socioeconomic disadvantage: systematic review

**DOI:** 10.3389/fpubh.2024.1332720

**Published:** 2024-02-19

**Authors:** Himal Singh, Florence Samkange-Zeeb, Jonathan Kolschen, Ruben Herrmann, Wiebke Hübner, Núria Pedrós Barnils, Tilman Brand, Hajo Zeeb, Benjamin Schüz

**Affiliations:** ^1^Institute of Public Health and Nursing Research, University of Bremen, Bremen, Germany; ^2^Department of Prevention and Evaluation, Leibniz Institute for Prevention Research and Epidemiology – BIPS, Bremen, Germany; ^3^Health Sciences Bremen, University of Bremen, Bremen, Germany

**Keywords:** health literacy, socioeconomically disadvantaged, intervention strategies, multilingual education, face-to-face, albatross plots, systematic review

## Abstract

**Background:**

Experiencing financial insecurity and being underserved is often associated with low health literacy, i.e., the ability to identify, obtain, interpret and act upon health information, which may result in poor health outcomes. Little is known about effective interventions for promoting health literacy among underserved populations. The objective of this systematic review is to summarize the literature on such interventions and identify characteristics that differentiate more effective interventions.

**Methods:**

Following PRISMA guidelines we searched the databases SCOPUS, Pubmed, Web of Science core collection and CINAHL. We included primary studies with a quantitative study design and control groups testing interventions to increase health literacy or health knowledge in underserved populations between 18 and 65 years. Where possible, we converted effect sizes into Cohen’s d and compared mean differences of intervention and control groups. Albatross plots were created to summarize the results according to different health literacy and health knowledge outcomes.

**Results:**

We screened 3,696 titles and abstracts and 206 full texts. In total, 86 articles were analyzed, of which 55 were summarized in seven albatross plots. The majority of the studies (*n* = 55) were conducted in the United States and had a randomized controlled study design (*n* = 44). More effective intervention approaches assessed needs of participants through focus group discussions prior to conducting the intervention, used bilingual educational materials, and included professionals fluent in the first languages of the study population as intervention deliverers. Additionally, the use of educational materials in video and text form, fotonovelas and interactive group education sessions with role playing exercises were observed to be effective.

**Discussion:**

Although the outcomes addressed in the included studies were heterogeneous, effective intervention approaches were often culturally sensitive and developed tailored educational materials. Interventions aiming to promote health literacy in underserved populations should hence consider applying similar approaches.

**Systematic review registration**: https://www.crd.york.ac.uk/prospero/display_record.php?RecordID=323801, PROSPERO registration ID: CRD42022323801.

## Introduction

Health literacy (HL) can be defined as a person’s “knowledge, motivation, and competences to access, understand, appraise and apply health information in order to make judgments and take decisions in everyday life concerning healthcare, disease prevention and health promotion” (1).

HL is an important resource when people engage with health-related information or the healthcare system. At the same time, low levels of HL are a risk factor for numerous health and health behavior outcomes. For example, individuals with low levels of HL suffer from higher mortality rates, have lower participation in cancer screening programs and show lower levels of preventive behaviors in general than those with higher HL (2–4).

Lower levels of HL are associated with difficulties taking medications as recommended [e.g., using non-standardized dosing tools (5), or recognizing all medications (6)]. Persons with low HL levels display more problems understanding health messages and more difficulties interpreting medication and nutrition labels (7–9).

This, however, does not necessarily imply individual deficits, but deficits in the way of how health-related information is presented or healthcare providers communicate (10).

HL levels are distributed unequally along social strata (11), and in particular population groups that face socioeconomic disadvantages^1^ are at high risk for low HL. This includes individuals with low educational attainment, unemployed people (see Footnote 1), people with a migration background, and in general people with a low socioeconomic status (12–15). In Europe, 74% of the group with a very low socioeconomic status show limited HL compared to 47% of the general population (11).

These studies indicate an urgent need to support and promote HL, especially in financially insecure and underserved populations. However, surprisingly little is known about effective interventions for promoting HL both in underserved populations and in general, and about potentially modifiable determinants of HL that could form the basis of interventions. A recent rapid review identified the following as potentially modifiable determinants of HL in working-age populations: providing accessible health information in digital and analog media, support in multiple and potentially simplified languages and health behaviors (16). However, it is not clear whether these strategies work with financially insecure and underserved populations.

There are several systematic reviews that summarize the effects of HL interventions on specific health outcomes or in specific populations (3, 17–19). However, there is a dearth of reviews on the effects of HL-focused interventions on actual HL outcomes (such as functional HL or mental health literacy). This is not only an academic issue, but crucial in order to understand which intervention features are most likely to promote HL. As HL is an important determinant of interactions with health services, health behaviors, and health outcomes (2–4), more knowledge on how to successfully intervene on HL is needed.

Although the definition of HL comprises the four dimensions accessing, understanding, appraising and applying health information, we also included health knowledge outcomes because this is a major part of HL and is decisive in implementing health promoting health behavior (20). This was highlighted during the COVID-19 pandemic, where health knowledge predicted protective behaviors, such as wearing masks, social distancing, and washing hands (21–23). Also, health knowledge might serve as a proxy for how well people access and understand health information.

This systematic review therefore aims to summarize existing interventions with the goal to identify effective strategies and techniques to promote HL (primary outcome) and health knowledge (secondary outcome) among working-age populations experiencing socioeconomic disadvantage.

The age group was chosen as we focused on people who could be potentially unemployed (in other words working-age populations) since under-employed people, unemployed people and especially long-term unemployed people often fulfill multiple risk factors for low HL such as financial insecurity, a migration history or limited literacy skills (24–26).

## Methods

The systematic review was conducted in line with the PRISMA guidelines. The respective protocol was registered in May 2022 at PROSPERO (registration number: 323801).

### Eligibility criteria and information sources

Four databases were searched in April 2022: SCOPUS (via Elsevier), Pubmed (via Ovid), Web of Science core collection and CINAHL (via EBSCO). The references of the included articles were searched for further relevant literature.

Inclusion criteria:

- Quantitative study design including at least an intervention and a control group.
- Primary or secondary outcome of the study has to be any kind of health literacy or health knowledge outcome.
- Focusing on financially insecure and underserved working-age populations between 18 and 65 years including migrant populations (e.g., adults living in deprived areas, unemployed, low-income, poor, precarious employment status, undocumented legal status, low educational attainment, low literacy, low socioeconomic status).

Exclusion criteria:

- Articles not reporting HL as an outcome.
- Articles not published in English or German.
- Articles not reporting any statistical tests or pre-and post-results.
- Articles where the full-text could not be accessed.
- Articles not reporting the mean age of participants (excluded from the main analysis, but included in sensitivity analysis).

### Search strategy

As recommended for systematic reviews, we used the PICO approach (Among working-age populations experiencing socioeconomic disadvantage (P) which health literacy interventions (I) compared to other or no such interventions (C) are effectively improving any health literacy and/or health knowledge outcome (O)?). The following search and MESH terms were used: “unemployed,” “redundant,” “unoccupied,” “refugees,” “low socioeconomic status,” “disadvantaged,” “deprived,” “low socioeconomic,” “poor,” “poverty,” “homeless,” “underprivileged,” “precarious employment status, “undocumented people,” “migrant,” “immigrant” (population); “intervention,” “health campaign,” “education” (intervention); “controlled study,” “controlled trial,” “control group,” “comparison group,” “reference group,” “experiment,” “quasi-experiment” (comparator); and “health literacy” or “health knowledge.” The search results were limited to adult populations by excluding the terms “children” “and adolescents.” Populations with a mean age of over 65 years were excluded during title/abstract and full-text screening. The search strategies for all searched databases can be found in Supplementary file 1.

### Selection of studies

All articles identified from the searched databases were imported to the reference management software Endnote, where duplicates were removed. The remaining articles were then exported to the systematic review software Rayyan (27). Further duplicates missed during the initial deduplication were identified and removed in Rayyan before beginning the title and abstract screening. The title and abstract screening and full-text screening were done independently by two authors (FSZ & HS). All conflicts were resolved by discussion until a consensus was reached.

### Data extraction

All data items were extracted twice by four authors independently in two-party teams (JK & FS and RH & HS) based on the extraction table devised *a priori* and presented in the study protocol (PROSPERO registration ID: CRD42022323801). The results of the extracted items were compared and conflicts were double-checked in the original article.

Among others, the following information was extracted: bibliographic information, study design, description of the intervention and control condition, definition of exact outcome, methods of outcome measurement, and effect estimator as reported in the article (Supplementary file 2).

### Risk of bias analysis

Risk of bias was assessed independently by two authors (HS & JK) using the Risk of Bias tool (RoB) 2 of Cochrane for randomized controlled trials (RCT) (28) and the ROBINS-I for non-randomized controlled trials and quasi-experimental trials (29). Discrepancies in the assessments were discussed until resolved.

### Data synthesis and analyses

Extracted study data were initially reviewed narratively. To begin with, we grouped the different relevant primary and secondary outcomes reported in the included studies in two categories as follows: (i) Health literacy: Functional health literacy and mental health literacy as primary outcomes; (ii) Health knowledge: cancer screening knowledge, child feeding knowledge, diabetes knowledge, food knowledge and HIV knowledge as secondary outcomes.

Due to heterogeneity in study outcomes, limitations in reporting, and variation in study designs, we refrained from conducting formal meta-analysis and used albatross plots to summarize findings. Albatross plots are a statistical visualization technique that can be used as an alternative to narrative reviews or to summarizing techniques such as vote counting in case of high heterogeneity of primary studies, as they allow comparing the approximate sizes of the effects and take into account the sample sizes of the primary studies as long as *p*-values are provided. In principle, albatross plots are scatter plots of study sample sizes against two-sided *p*-values and effect directions (in our case: favors HL intervention or favors control; (30)). Small two-sided *p*-values favoring the control group appear on the far left side of the panel, and small two-sided p-values favoring the HL intervention on the right side. Studies plotted close to the center have very small or non-significant effects. In addition, lines are added to the plot (albatross wings) that represent different levels of effect sizes (in our case: *d* values) based on the different alpha levels (0.05, 0.01 and 0.001) derived from primary study data. For a more detailed discussion of obtaining estimates for the effect size lines, see Harrison et al. (30).

For the comparison of different modes of delivery, we additionally indicated whether a study was conducted face-to-face or not by using different symbols in the plot.

We extracted sample sizes, *p*-values and effect sizes to summarize the intervention effects and to create seven albatross plots according to the different HL and health knowledge outcomes reported in the included studies. We compared mean differences (or differences between respondents giving correct and wrong answers in pre-and post-surveys for the intervention and control groups, respectively) to compute effect sizes. Where possible, all effect sizes reported in the studies (e.g., odds ratios, means of intervention and control groups, t values, etc.) were transformed into Cohen’s d. Only studies providing information regarding, sample size, *p*-values and effect sizes were included in the albatross plots. Effect sizes were calculated if not given insofar as mean, sample size and standard deviation were given. Sample sizes, p-values, and Cohen’s d of all included studies considered in the albatross plots are provided in Supplementary file 3. Albatross plots were created via Stata 17.0.

### Sensitivity analysis

Sensitivity analysis was conducted by excluding articles that did not specifically report the study populations’ mean age or age range. Most of these excluded articles reported age group distributions of the study population.

## Results

**Figure 1:**
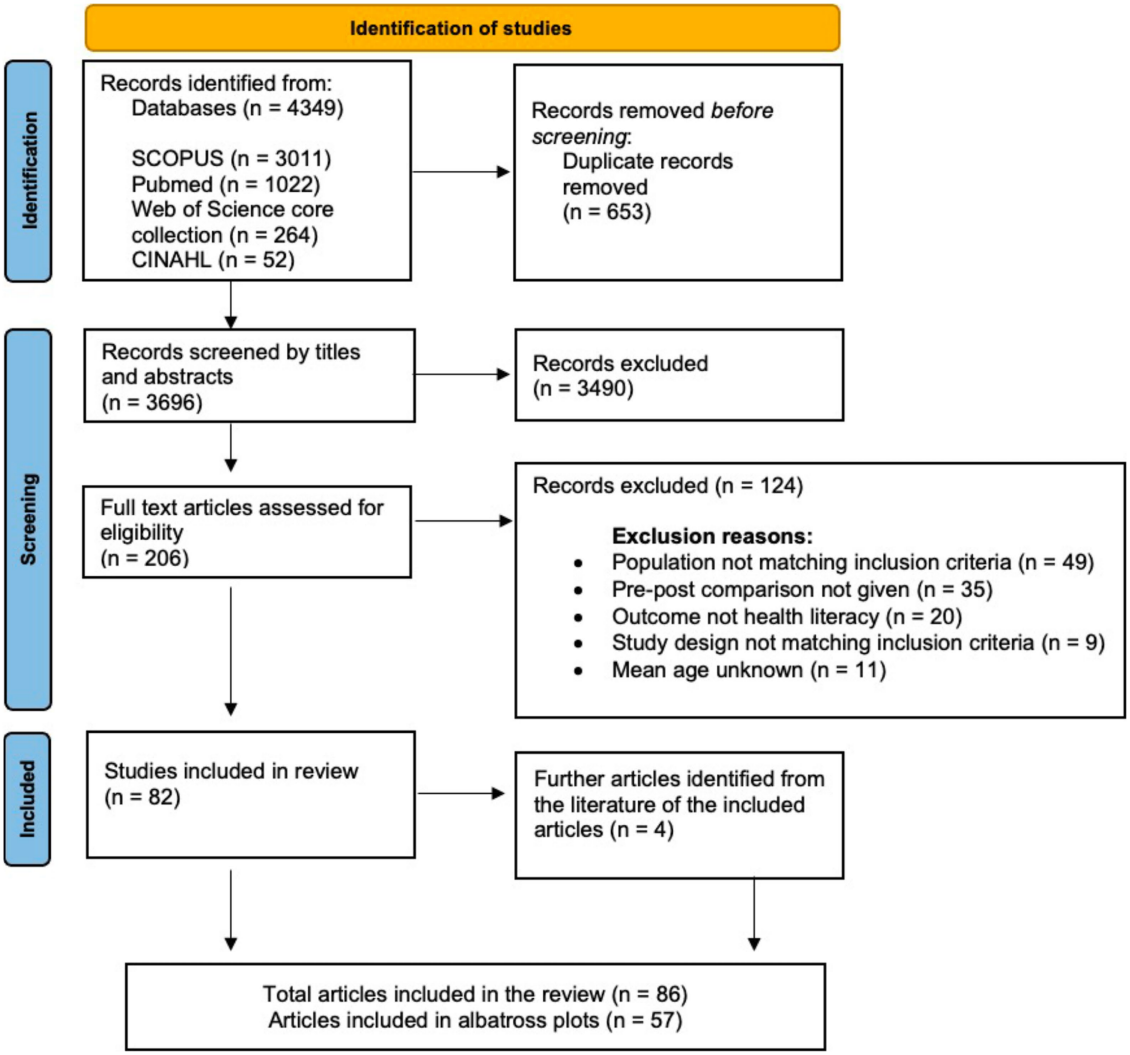
PRISMA flow chart of the search process.

We screened 3,696 titles and abstracts and 206 full-texts. 82 articles met eligibility criteria and were included in the review. Four further articles were identified through hand-searching the literature of the included articles. Hence, in total, 86 articles were included in the main analyses, and 57 articles were included in albatross plots (Figure 1).

The main reasons for exclusion in the full-text phase were population not matching inclusion criteria (*n* = 49), no pre-post comparisons reported in the analyses (*n* = 28), no health knowledge outcome (*n* = 20), or the study design not matching the inclusion criteria (*n* = 9).

### Study characteristics

The 86 studies included in the review were conducted in 20 different countries. The majority were conducted in the United States (*n* = 55), followed by China (*n* = 5), Taiwan (*n* = 4) and South Korea (*n* = 3).

51% of the included studies (*n* = 44) had a randomized controlled study design (of which 16 were cluster randomized controlled trials), while the remainder had a randomized or non-randomized quasi-experimental (*n* = 33), or another non-randomized controlled study design with a pre-post comparison (*n* = 9; Supplementary file 2).

All included studies were written in English and were published between 1990 and 2021. All study populations were working-age (18 to 65 years) and were potentially underserved or financially insecure. Mostly, the study populations were recruited from low-income neighborhoods and included migrants, young mothers, or clinical patients (with a low socioeconomic status) with pre-existing diseases such as diabetes and HIV. All in all, demographics of the study populations were roughly comparable (Supplementary file 2).

### Health literacy outcomes and instruments

The included studies assessed a broad range of HL and health knowledge outcomes, including functional HL, mental health literacy, cancer screening knowledge, child feeding knowledge, diabetes knowledge, food knowledge, and HIV knowledge (Table 1; Supplementary files 2, 3).

**Table 1:** Summary of review findings.

Interventions to promote health literacy among resource-limitedly disadvantaged populations				
Population: Financially insecurely disadvantaged populations between 18 and 65 years				
Outcomes	Comparison of studies		Total no. of participants (studies)	Risk of bias overall
	Studies that found significant improvements in health literacy or health knowledge (%) Risk of bias of individual studies	Studies that did not find significant improvements in health literacy or health knowledge (%) Risk of bias of individual studies		
Functional health literacy	2 (50%) (212 participants) - -	2 (50%) (311 participants) o -	563 (4 studies)	High risk of bias in 3/4 studies
Mental health literacy	3 (75%) (390 participants) o - -	1 (25%) (20 participants) -	410 (4 studies)	High risk of bias in 3/4 studies
Cancer screening knowledge*	3 (60%) 897 participants o - -	2 (40%) 390 participants o -	987 (4 studies)	High risk of bias in 3/4 studies
Childfeeding knowledge*	9 (60%) 6,810 participants - o - o - o - o -	6 (40%) 3,044 participants - - o o o o	8,007 (12 studies)	High risk of bias in 6/12 studies
Diabetes knowledge	1 (25%) 72 participants -	3 (75%) 511 participants o + o	583 (4 studies)	High risk of bias in 1/4 studies
Food knowledge*	10 (67%) 4,093 participants - - - o – o o + − 0	5 (33%) 1,023 participants - o o - o	4,329 (12 studies)	High risk of bias in 6/12 studies
HIV knowledge*	12 (67%) 3,532 participants - o - - o - - - - - - -	6 (33%) 2067 - o - - - -	6,515 (17 studies)	High risk of bias in 14/17 studies

**Figure 2:**
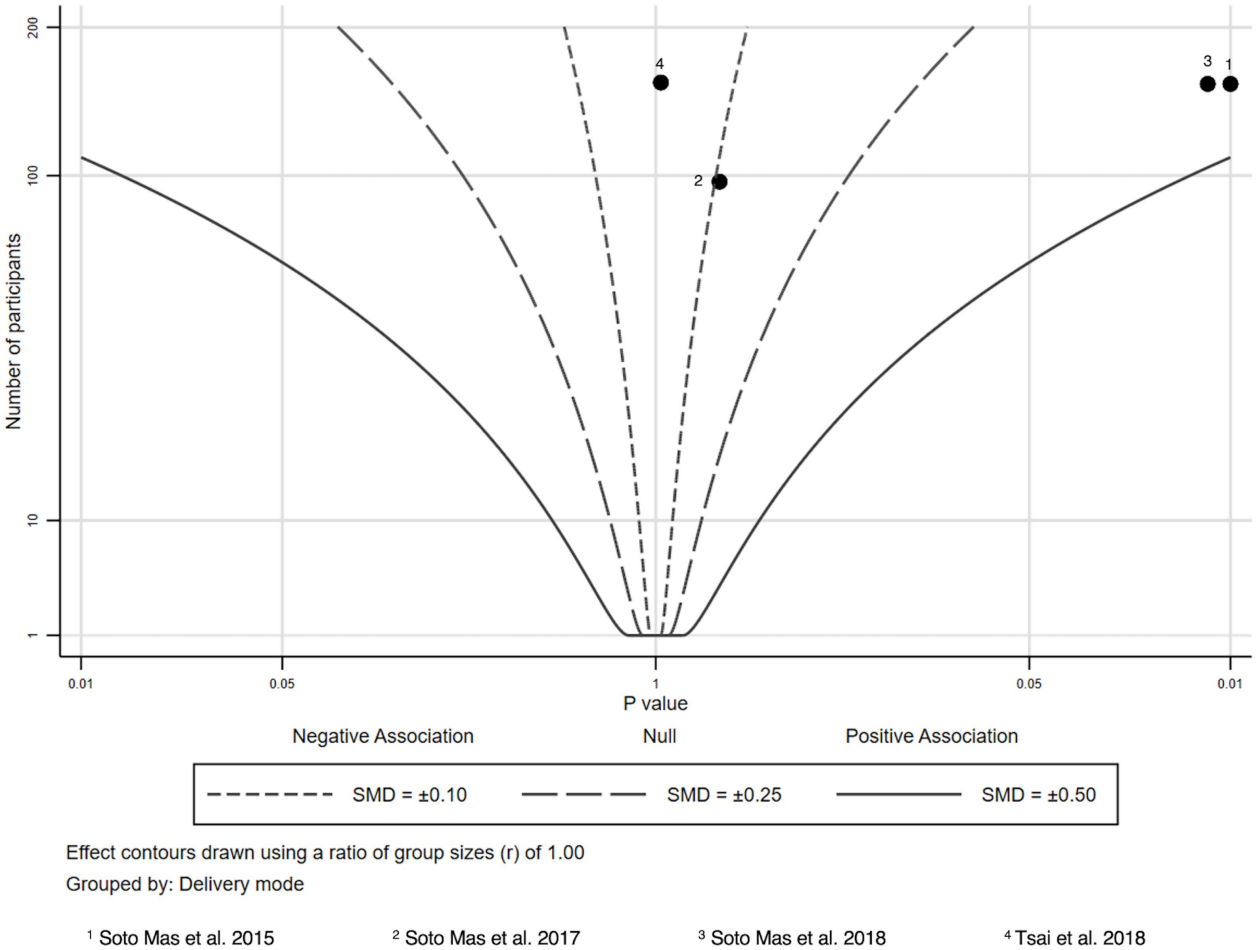
Albatross plot—functional health literacy.

**Figure 3:**
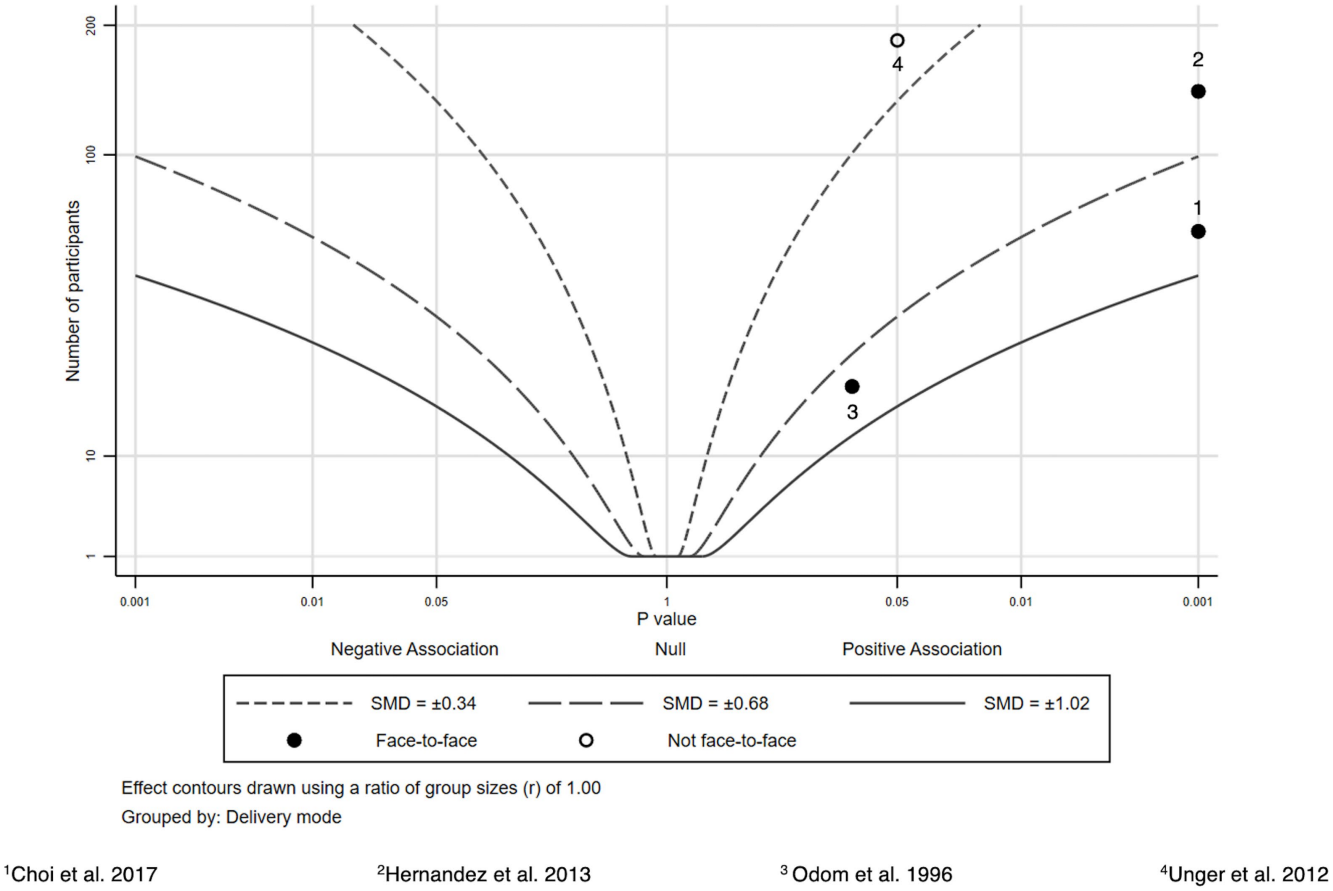
Albatross plot—mental health literacy.

**Figure 4:**
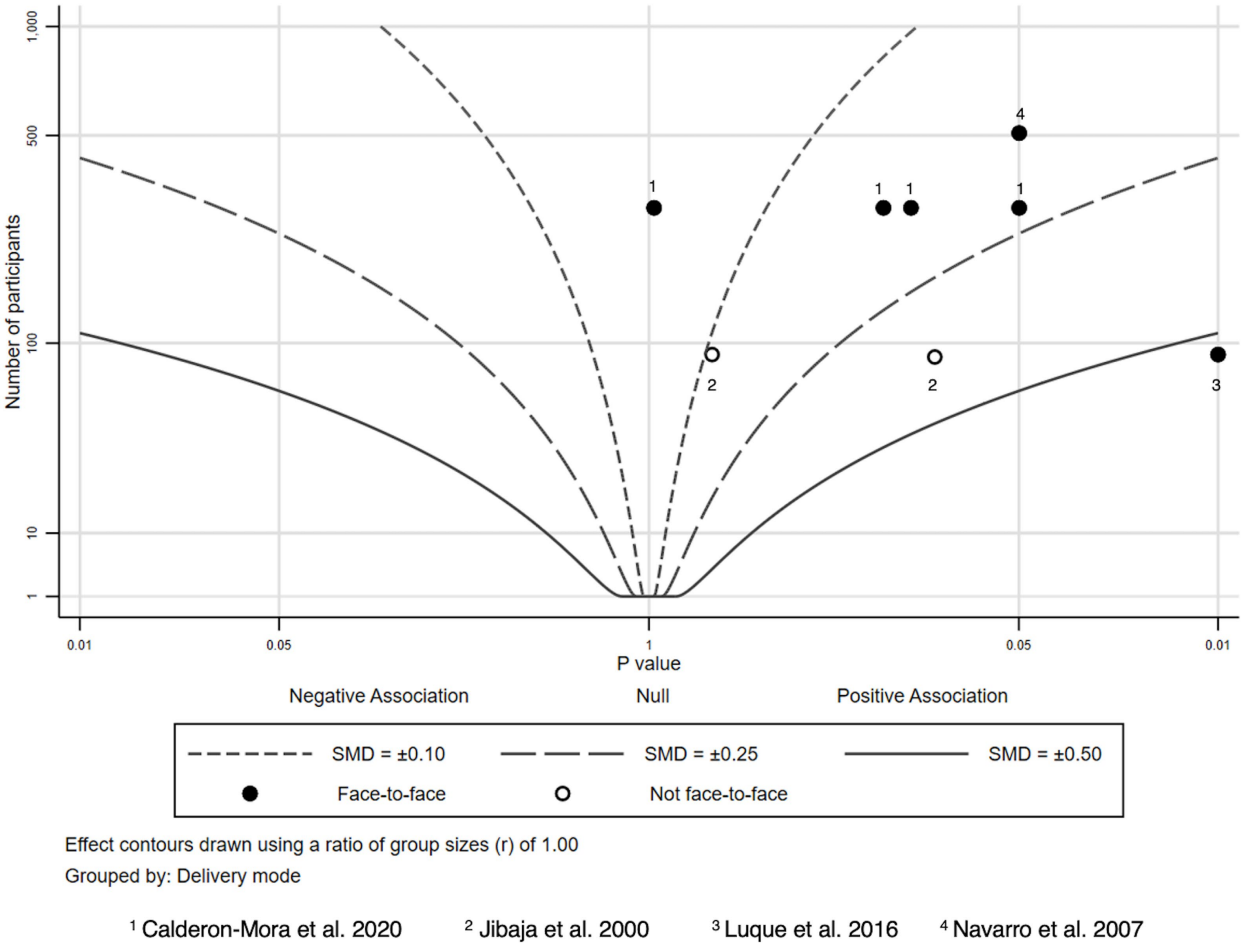
Albatross plot—cancer screening knowledge.

**Figure 5:**
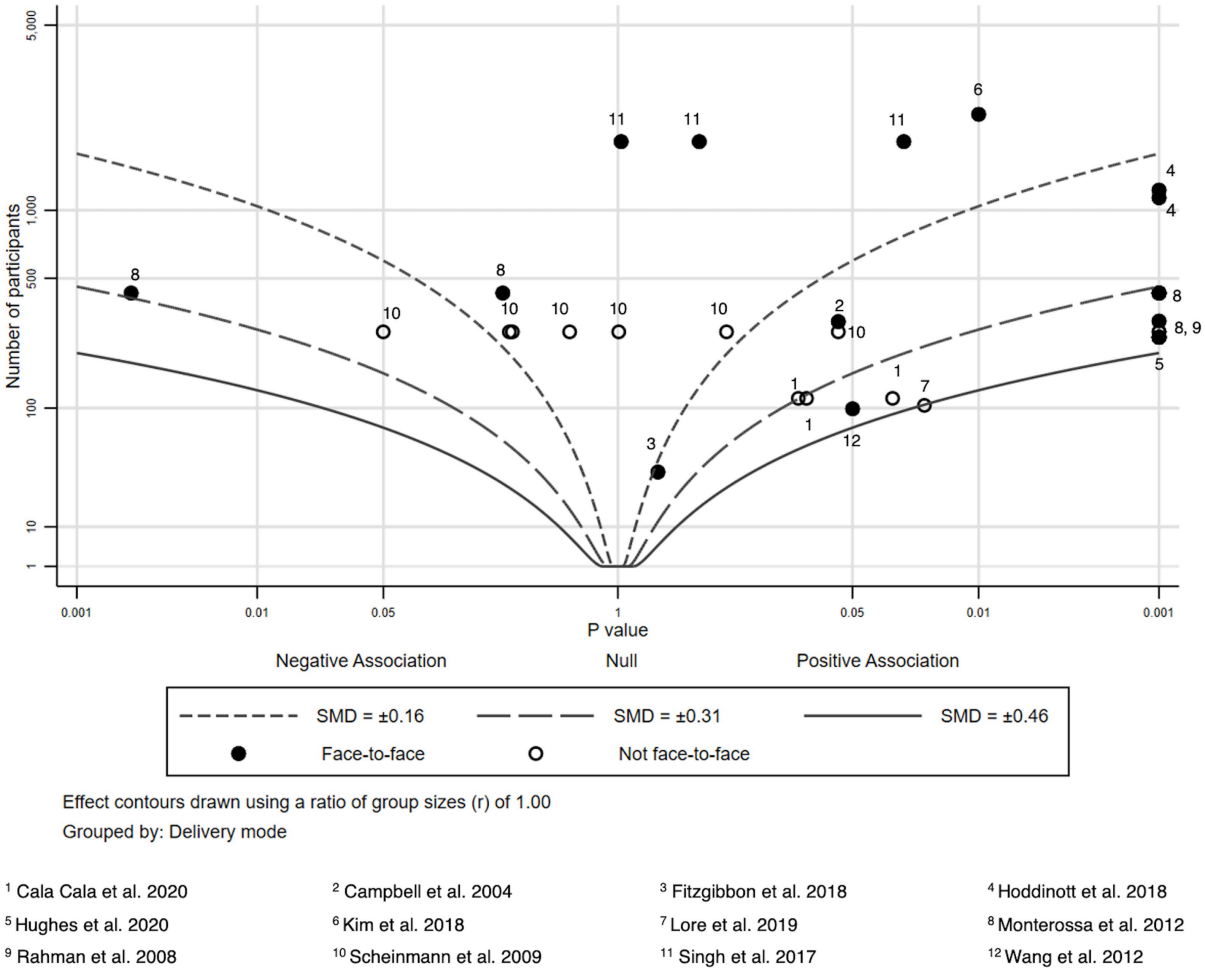
Albatross plot – Child feeding knowledge.

**Figure 6:**
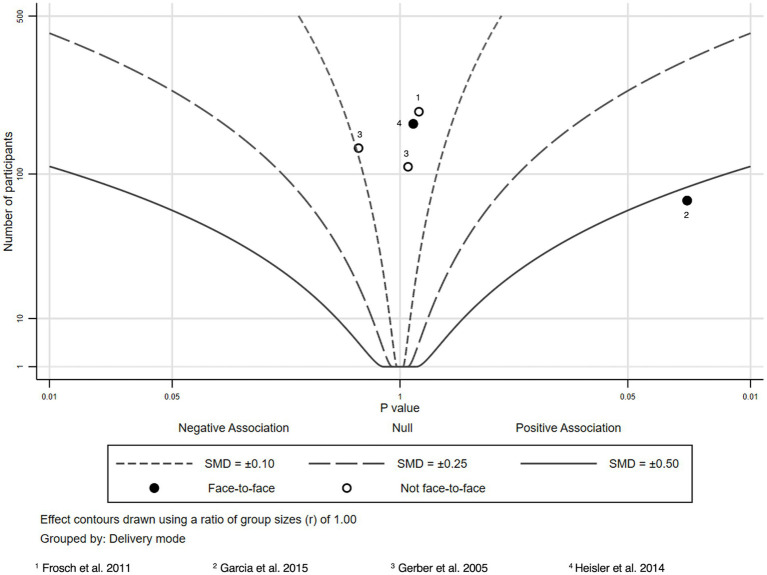
Albatross plot—diabetes knowledge.

**Figure 7:**
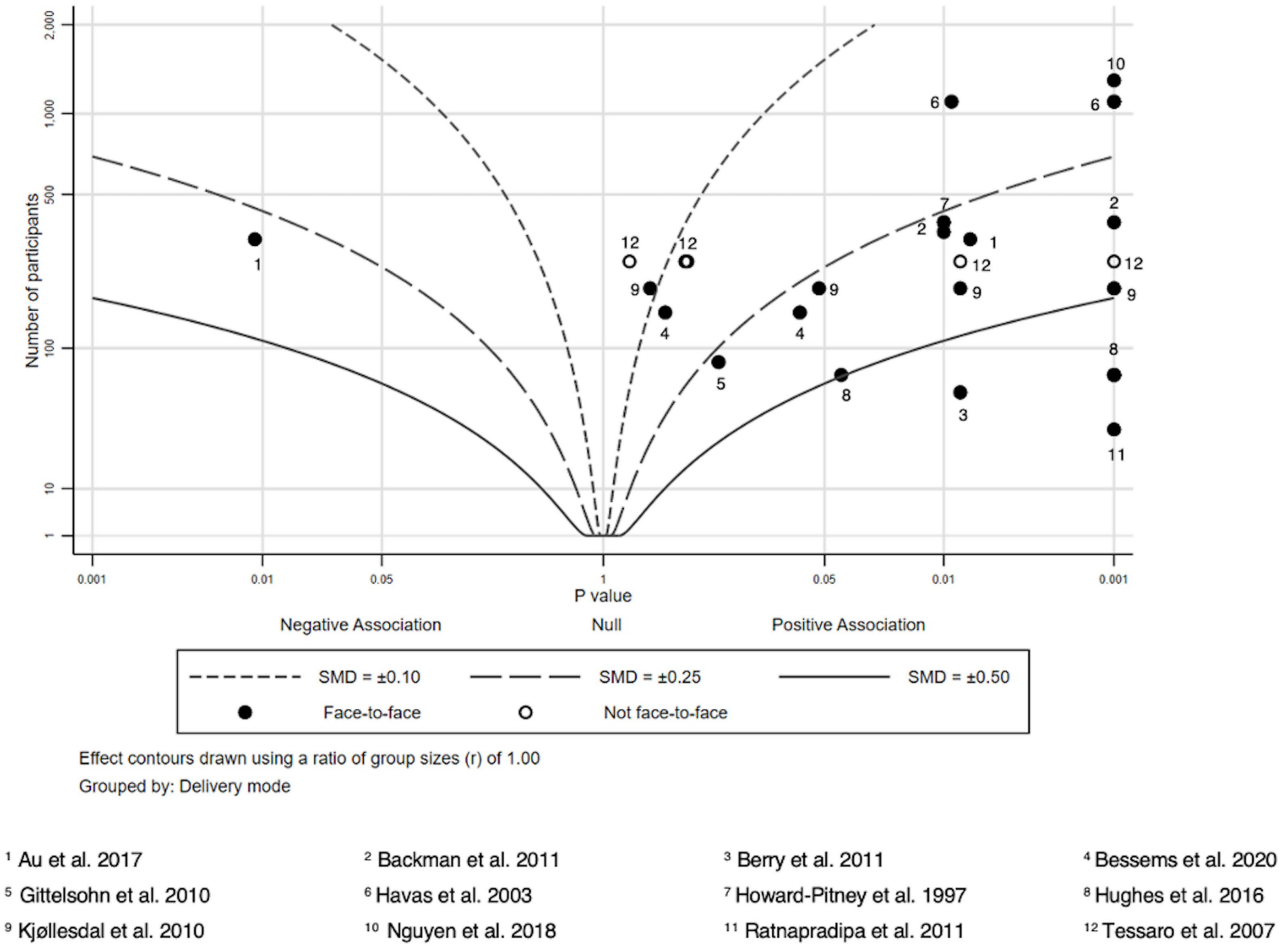
Albatross plot—food knowledge.

**Figure 8:**
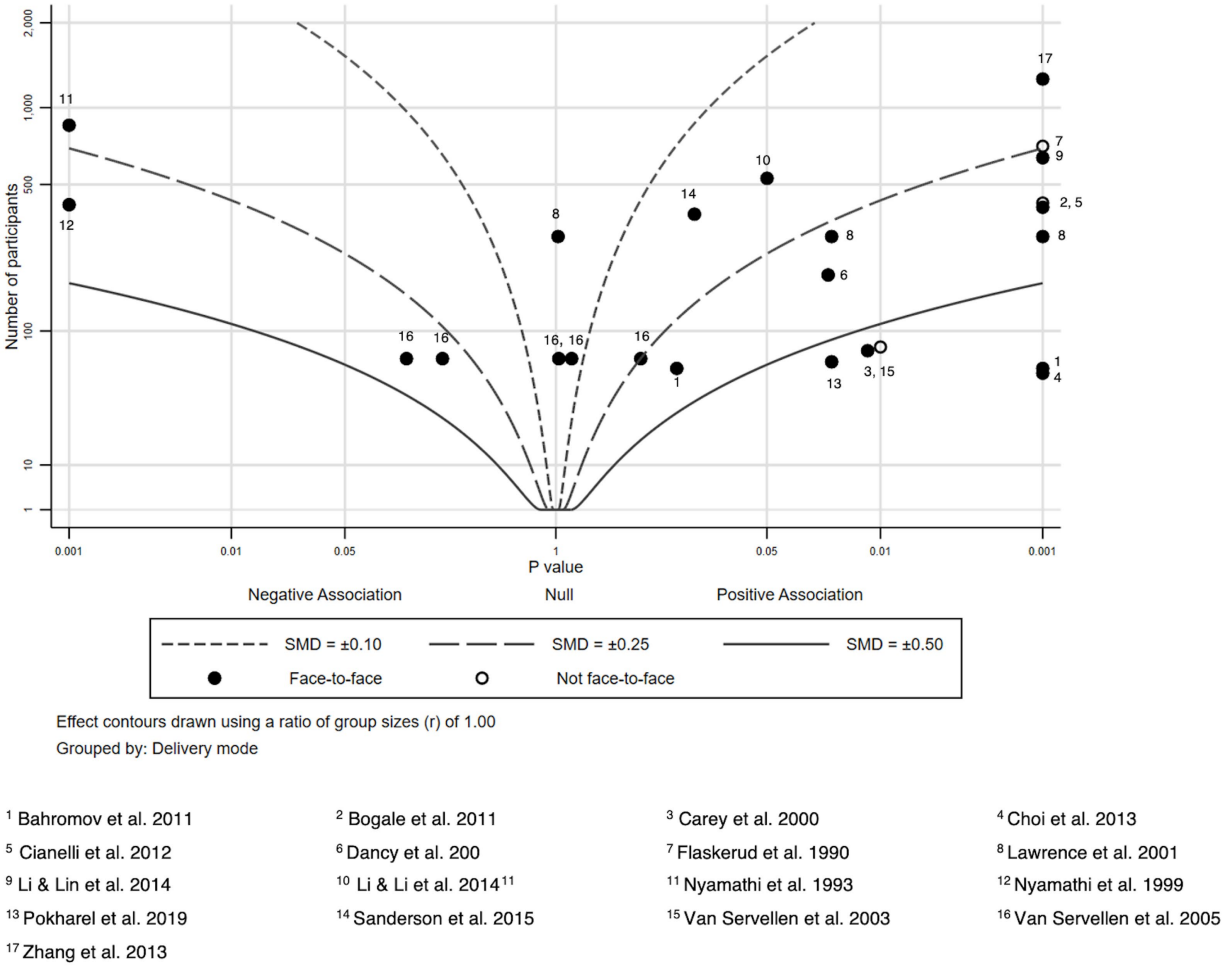
Albatross plot—HIV knowledge.

- High risk of bias, o Moderate risk of bias, + Low risk of bias. *Some studies included multiple outcomes and appear in both rows: Statistically significant health knowledge improvement and no statistically significant health knowledge improvement (Supplementary file 3). (Summary of risk of bias displayed in same order as studies labeled in Figures 2–8).

Mostly, self-developed tools were used to measure the respective outcomes. Some validated tools used in the included studies were the TOFHLA/S-TOFHLA for functional HL, (31–33) the HIV knowledge Questionnaire for HIV knowledge (34, 35) or the Food Frequency Questionnaire (36) (Supplementary files 2, 3).

### Intervention effects by outcome and intervention strategies applied

#### Functional health literacy

We analyzed the results of four studies reporting functional HL (31–33, 37) (Figure 2). All studies included a face-to-face component and two studies showed a statistically significant improvement in functional HL scores (32, 33).

In both studies, Spanish-speaking adults received curriculums focusing on improving HL (32, 33). The curriculum included components based on theories of HL, adult learning principles and sociocultural theories of literacy and communication and was designed for adults with limited English language skills (32). In one of the studies, participants additionally received cardiovascular-specific content (33).

### Mental health literacy

Four studies reporting mental health literacy outcomes were included (38–41) (Figure 3). Mental health literacy scores improved statistically significantly in three of these, all of which had a face-to-face component (38, 39, 41).

The interventions comprised mental health improvement services by bilingual gatekeepers following a multilingual mental health improvement guidebook developed by authors in eight different languages (38) or fotonovelas, a form of entertainment education aiming to promote depression and mental health literacy (39, 41). Both Unger et al. and Hernandez and Organista implemented the fotonovela “Secret Feelings” (42) which was presented in English and Spanish at a 4^th^ grade reading level and describes the story of a Hispanic wife and mother who deals with depression and eventually decides to obtain counseling and medication. Using dialogs and photographs the fotonovela informs about depression symptoms and the use of antidepressants.

### Cancer screening knowledge

We found four studies providing eight effect sizes and tests addressing knowledge about cancer screening (43–46) (Figure 4). In three of these tests, the intervention group scored significantly better than the control group (43, 45, 46). All significant tests were reported for face-to-face interventions, the one study without a face-to-face component yielded no significant effect (44).

Effective intervention approaches to increase cancer screening knowledge incorporated bilingual gatekeepers delivering educational sessions consisting of informational handouts, message cards addressing barriers to screening, a short-animated video, in-class activities and informational brochures (43, 45). In another study, trained community health advisors, “*consejeras”* provided interactive educational sessions in their communities (46).

### Child feeding knowledge

We analyzed 12 studies which provided 35 effect sizes regarding child feeding practices and maternal nutrition (47–58) (Figure 5). In 22 tests from 9 studies, participants from the intervention groups showed better knowledge scores compared to participants of the control groups (47, 50–55, 57, 58). In two tests an inverse association was shown with participants of the control groups showing better knowledge scores (54, 56). In the remaining outcomes, the intervention groups tended to score better, however, the improvements compared to the control groups were not statistically significant.

Studies with a larger effect mostly had a face-to-face component.

Components of the intervention programs showing larger effects were bilingual educational materials in form of DVDs and booklets (47), bilingual video-based learning sessions with African-American mothers and mothers with Hispanic backgrounds talking about challenges and evidence-based methods while feeding their children (51), scripted messages developed based on focus group discussions which were delivered by nurses and at clinics or home visits (54), and a 6-month computer-based curriculum consisting of 12 modules implemented through weekly home visits in one-on-one educational sessions with parents (53). Using bilingual language materials was a key part of the described interventions above.

### Diabetes knowledge

Four studies and five outcomes on diabetes knowledge (Figure 6) (59–62) were identified through our searches.

Intervention recipients tended to show better knowledge, however, only one (pilot) study resulted in statistically significant knowledge improvements (60). Here, participants received in-home educational and behavioral modification sessions by registered nurses addressing among other topics, symptoms awareness and appropriate treatments for diabetes. The education session was followed by biweekly support telephone sessions.

### Food knowledge

12 studies and 30 effect sizes were analyzed related to food knowledge (36, 63–73) (Figure 7). In 20 tests from 10 studies, intervention recipients obtained better food knowledge scores compared to the control group (36, 63–65, 68–73). For one outcome, a negative association was shown, with the control group scoring higher than the intervention group (63). The remaining studies did not report significant intervention effects. Most studies with a positive intervention effect reported effect sizes above *d* = 0.25, partly above *d* = 0.5.

Successful intervention designs incorporated children teaching their parents in their native language about safe food handling knowledge (72), used community settings such as churches to distribute bilingual educational materials, organized activities such as cooking demonstrations and taste testing, provided weekly motivational interviewing sessions by telephone (69), or conducted group education sessions using culturally adapted materials (70). Similarly, a multicomponent 12-week weight management program based on focus groups and designed to be culturally sensitive to Hispanic culture was effective. Nutrition classes were set up according to the needs of participants aiming to keep cooking their family recipes and providing advice on how to make them healthier. Another part of the intervention was exercise education and exercise classes including stretching, walking, cardio and kickboxing, some of which could easily be done at home (65).

### HIV knowledge

We analyzed 17 studies and 24 effect sizes regarding HIV knowledge (34, 35, 74–88) (Figure 8). In 15 outcomes from 15 studies HIV knowledge of intervention receivers improved significantly compared to the control groups (34, 35, 74–81, 84–86, 88).

A negative association was shown in two studies where the control group scored better (82, 89). In the remaining studies, there were some non-statistically significant improvements in the intervention groups.

Interventions with larger effects included 4-week educational sessions in video and text form (76), an intervention program delivered on a train and consisting of didactic presentation, role-playing, and group discussions (74) and an educational intervention using motivational interviewing strategies based on information-motivation behavioral skills model (35).

A key part of the interventions was the translation of intervention materials into the respective first language of the study participants, or intervention deliverers being fluent in the study participants’ first language (74, 76).

### Risk of bias analysis

**Figure 9:**
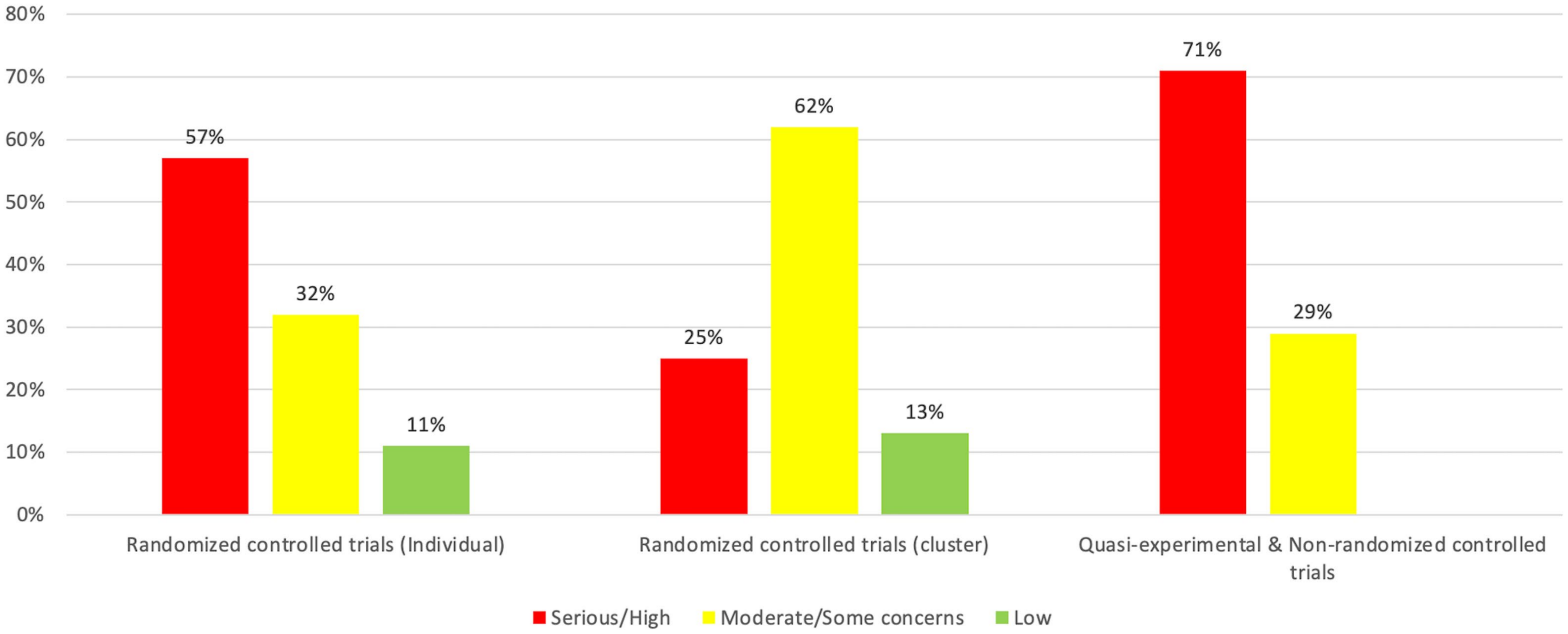
Risk of bias analysis.

We assessed the risk of bias in a variety of study designs using different tools. Due to poor reporting and lack of information provided, several studies were rated as low-quality studies. According to the RoB 2 from Cochrane, 11% (*n* = 3) of individually randomized controlled trials were given a low-risk rating, 32% (*n* = 9) were categorized as having some concerns and for 57% (*n* = 16) a high risk of bias was assessed. For cluster randomized controlled trials, 13% (*n* = 2) were rated as low-risk, 62% (*n* = 10) had some concerns and 25% (*n* = 4) were classified as high risk. While employing the ROBINS-I tool, 29% (*n* = 12) of quasi-experimental and non-randomized controlled trials were considered to have a moderate risk of bias, while 71% (*n* = 30) were assessed as having a serious or critical risk of bias (see Figure 9 and details in Supplementary file 4).

In an additional analysis, we assessed the included studies based on their risk of bias. We reran the albatross plots and compared effect sizes of studies with higher and lower risk of bias. Studies with a higher risk of bias more often showed larger effect sizes (details in Supplementary file 5).

### Sensitivity analyses

11 articles not specifically reporting the study populations’ mean age or age range were analyzed separately and the results were compared to the main findings. Similar as in the main analysis most of the studies considered for the analysis were conducted in the United States (*n* = 4). Further studies were conducted in Australia, Bangladesh, China, Kenya, Nigeria, Netherlands and Turkey. Effective intervention approaches to improve health knowledge delivered educational programs via home visits (90), made use of different delivery modes including lectures, role-plays and interactive sessions (91) and incorporated bilingual gatekeepers as intervention deliverers (92) (Details in Supplementary file 6).

## Discussion

Health literacy (HL) is distributed unequally. Underserved and financially insecure groups often report low levels of HL which in turn has a negative impact on their health and interactions with health services. Finding effective ways to improve HL in financially insecure and underserved populations is therefore of high public health relevance. To our knowledge, this is the first systematic review to assess the effects of interventions aiming to improve HL or health knowledge in working-age population groups experiencing socioeconomic disadvantage.

We found that successful intervention approaches (i.e., those that led to meaningful improvements in HL or health knowledge compared to the control groups) often incorporated a face-to-face component (31, 32, 35, 41, 51, 53–55, 60, 65, 69, 70, 72–74, 80) and often used culturally sensitive and multilingual materials with intervention deliverers being fluent in the first languages of the addressed populations, e.g., children of the study participants or bilingual gatekeepers (31, 32, 46, 47, 51, 65, 69, 70, 72).

### Language support and health literacy

Language support in the majority language is also crucial for programs targeting non-native speakers. Effective studies for example implemented an English as a Second Language Curriculum combining HL content and English language instructions while using bilingual education material (32). Here, the intervention combined language-related content to improve English language proficiency through listening, reading and speaking exercises with health-literacy-related content to improve navigation of the participants within the healthcare system as well as numeracy skills and skills related to preventive practices.

The results of our review support the existing evidence on the role of language proficiency as a component of effective HL interventions. A 2018 review concluded that HL intervention approaches should be designed according to the specific needs of the target groups and use easy-to-understand language (93). An intervention (94) designed for unemployed minority or migrant women illustrates the important role of language proficiency for disadvantaged populations: Here, a key finding was that women from similar cultural and linguistic backgrounds were essential to approach and retain the target group in the intervention and consequently improve HL.

Similar strategies seemed to be effective in improving MHL and other health knowledge outcomes analyzed in the review. Successful MHL promotion interventions used a face-to-face component (38, 39, 41), easy-to-understand language, bilingual gatekeepers, or fotonovelas which not only reduced text but were also created according to the needs of the study populations and with relatable characters (39, 41). Further key parts of successful intervention strategies included multilingual intervention materials and in-home education sessions. Focus groups guiding the design of the intervention were important to address the specific needs of the target population (47, 51, 60, 65, 69).

Together with our review, these findings suggest that low-threshold communication of the intervention contents and addressing culture-specific aspects are essential if HL interventions in disadvantaged populations are to succeed.

### Importance of face-to-face interventions

Interventions including face-to-face components seemed to be most effective in increasing HL and health knowledge. As virtually all alternatives to face-to-face delivery modes such as digital tools, self-help guides, etc. require resources and access (95–97), which in turn are distributed unequally across socioeconomic strata (96, 98), it is hardly surprising that face-to-face interventions are more effective in disadvantaged populations – alternatives could essentially be inaccessible. As digital literacy skills in particular are tied to socioeconomic factors such as higher education (99), this suggests that face-to-face components might be essential features for interventions that aim to be effective in underserved and financially insecure populations.

At the same time, low literacy needs to be distinguished from language proficiency. Migrant populations, e.g., might have high literacy skills in general but might not be language proficient in the majority language of their country of residence and accordingly score low on HL measures. In this case, online education materials might be useful as long as they are provided in the first language of the migrants. The literature suggests that online education can be as effective as face-to-face education in high-literate populations such as university students or health professionals (100, 101). This means that online interventions may be an option for high-literate migrant populations, but not for low-literate ones. At the same time, as our results suggest that face-to-face components might be more effective, language courses for migrant populations might be good settings for additional health literacy content.

### Strengths and limitations

To our knowledge, this is one of the first systematic reviews to specifically focus on interventions on HL in financially insecure and underserved populations. Our search strategy was comprehensive, nevertheless, we may have missed relevant studies, for example, those that investigated health literacy intervention approaches in the context of complex interventions. The outcomes of the reviewed studies were heterogeneous and difficult to compare. Thus, no meta-analysis could be conducted, but albatross plots were created instead. Only few studies examined HL or MHL as primary outcome, therefore the results need to be interpreted with caution. However, comparing the results of the secondary outcomes (health knowledge outcomes) to the primary outcomes effective intervention approaches employed similar intervention strategies such as involving bilingual gatekeepers and using multilingual materials. It should also be noted that due to lack of information given, a significant number of studies were found to exhibit a serious risk of bias, even though many of them were RCTs. Clearly, more high-quality intervention studies on health literacy with a focus on underserved and financially insecure populations are required or at least study methods need to be described in more detail. Consequently, results of this systematic review need to be interpreted respectively, despite of the large number of studies included.

This review summarizes various articles and their key results to identify the best strategies for improving HL and health knowledge and filling the gap in the literature.

Still, more interventions are needed to identify effective ways of increasing all facets of HL (access, understand, appraise and apply health information) as measured by the HLS-EU-Q16 for, e.g., rather than functional HL only.

## Conclusion and implications

This review found that effective HL intervention approaches were more likely to use face-to-face components and information matched to participants’ language skills and cultural backgrounds. Accordingly, future interventions should be designed face-to-face and according to the needs of the addressed population. Here, in particular, language proficiency and cultural aspects seem to be important. In practical terms, this could be ensured by involving target groups in needs assessments and intervention design, for example through focus group discussions before and throughout designing the intervention.

Furthermore, differentiating between *low literacy* and *lack of language proficiency* is crucial. While for example individuals with migration history might lack proficiency in the majority language, they might have high literacy levels in their first language and would therefore profit from native language health information, most likely regardless of the modality (brochures, video-based, face-to-face or not). For populations with low literacy however, written health information might not be suitable, and such information should be provided face-to-face or in visual formats. Depending on the composition of the respective target population, bilingual staff with fitting cultural backgrounds is important to approach and keep participants in intervention programs.

Regardless of individual literacy skills and language proficiency, all disadvantaged populations will profit from health services with low literacy requirements. Offering easy-to-understand, multilingual materials in places that are part of daily life, i.e., churches, sports clubs, mosques, barbers, and shopping centers, could be a way of reaching disadvantaged populations and increasing HL.

## Data availability statement

The original contributions presented in the study are included in the article/Supplementary material, further inquiries can be directed to the corresponding author.

## Author contributions

HS: Conceptualization, Formal analysis, Investigation, Methodology, Software, Writing – original draft, Data curation, Writing – review & editing. FS-Z: Conceptualization, Data curation, Methodology, Software, Writing – review & editing. JK: Data curation, Methodology, Software, Writing – review & editing. RH: Data curation, Writing – review & editing. WH: Writing – review & editing. NB: Formal analysis, Writing – review & editing. TB: Project administration, Writing – review & editing. HZ: Conceptualization, Project administration, Writing – review & editing. BS: Conceptualization, Formal analysis, Methodology, Project administration, Supervision, Writing – original draft, Writing – review & editing.

## Footnotes

^1^To promote inclusivity and avoid stigmas we have refrained in the following from using the terms “socially disadvantaged” and “socioeconomically disadvantaged,” opting instead for the expressions “financial insecurity” and “underserved.” Nevertheless, for the sake of article visibility, the original term is retained in the title and when referencing to the literature.
