# Supplementary material for: Interventions to promote health literacy among working-age populations experiencing socioeconomic disadvantage: systematic review
Source: Front Public Health. 2024 Feb 19;12:1332720. doi: 10.3389/fpubh.2024.1332720 (PMC10909862; doi:10.3389/fpubh.2024.1332720)
Supplement: Supplementary file 1 [file Data_Sheet_1.ZIP › Supplementary file 1_ Search strategy.docx]

# Supplementary file 1 – Search strategy

# Interventions to promote health literacy among socioeconomically disadvantaged working-age population groups: Systematic review

Himal Singh^1*^, Florence Samkange-Zeeb^2^, Jonathan Kolschen^1^, Ruben Herrmann^1^, Wiebke Hübner^2^, Núria, Pedrós Barnils^1^, Tilman Brand^2^, Hajo Zeeb^2,3^, Benjamin Schüz^1,3^

^1^Institute of Public Health and Nursing Research, University of Bremen, Bremen, Germany

^2^Department of Prevention and Evaluation, Leibniz Institute for Prevention Research and Epidemiology – BIPS, Bremen, Germany

^3^Health Sciences Bremen, University of Bremen, Bremen, Germany

Search string SCOPUS

( TITLE-ABS-KEY ( "unemployed*"  OR  "redundant*"  OR  "unoccupied*"  OR  "refugees*"  OR  {Low socioeconomic status}  OR  "disadvantaged*"  OR  "deprive*"  OR  "low-socioeconomic*"  OR  "poor*"  OR  "poverty*"  OR  "homeless*"  OR  "underprivileged*"  OR{precarious employment status}  OR  {Undocumented people}  OR  "migrant*"  OR  "immigrant*" )  AND  TITLE-ABS-KEY ( "intervention*"  OR  {Health campaign}  OR  "education*" )  AND  TITLE-ABS-KEY ( {Controlled study}  OR  {Controlled trial}  OR  {Control group}  OR  {Comparison group}  OR  {Reference group}  OR  "experiment*"  OR  {Quasi experiment} )  AND  TITLE-ABS-KEY ( {Health literacy}  OR  {Health knowledge} ) )

Search string Pubmed

(unemployed OR Redundant OR Unoccupied OR Refugees OR "Low socioeconomic status" OR Disadvantaged OR Deprived OR "low socioeconomic" OR Poor OR poverty OR Homeless OR Underprivileged OR "precarious employment status" OR "undocumented people" OR Migrant OR Immigrant) AND (Intervention OR "Health campaign" OR Education) AND ("Controlled study" OR "Controlled trial" OR "Control group" OR "Comparison group" OR "Reference group" OR Experiment OR Quasi experiment) AND ("Health literacy" OR "Health knowledge")

Web of Science core collection

Search string

(((TS=(“unemployed*” OR “redundant*” OR “unoccupied*” OR “refugees*” OR “Low socioeconomic status” OR “disadvantaged*” OR “deprive*” OR “low-socioeconomic” OR “poor*” OR “poverty*” OR “homeless*” OR “underprivileged*” OR “precarious employment status” OR “Undocumented people” OR “migrant*” OR “immigrant*”)) AND TS=(“intervention*” OR “Health campaign” OR “education*” )) AND TS=(“Controlled study” OR “Controlled trial” OR “Control group” OR “Comparison group” OR “Reference group” OR “experiment*” OR “Quasi experiment”)) AND TS=(“Health literacy” OR “Health knowledge”)

CINAHL

AB ( unemployed OR redundant OR unoccupied OR refugees OR “Low socioeconomic status” OR disadvantaged OR deprive OR “low-socioeconomic” OR poor OR poverty OR homeless OR underprivileged OR “precarious employment status” OR “Undocumented people” OR migrant OR immigrant ) AND AB ( “intervention*” OR “Health campaign” OR “education*” ) AND AB ( “Controlled study” OR “Controlled trial” OR “Control group” OR “Comparison group” OR “Reference group” OR “experiment*” AND AB ( “Health literacy” OR Health knowledge)
