# Supplementary material for: Interventions to promote health literacy among working-age populations experiencing socioeconomic disadvantage: systematic review
Source: Front Public Health. 2024 Feb 19;12:1332720. doi: 10.3389/fpubh.2024.1332720 (PMC10909862; doi:10.3389/fpubh.2024.1332720)
Supplement: Supplementary file 1 [file Data_Sheet_1.ZIP › Supplementary file 2_Articles included in the review and data extraction table.docx]

# Supplementary file 2 – All articles included in the systematic review and data extraction table

# Interventions to promote health literacy among socioeconomically disadvantaged working-age population groups: Systematic review

Himal Singh^1*^, Florence Samkange-Zeeb^2^, Jonathan Kolschen^1^, Ruben Herrmann^1^, Wiebke Hübner^2^, Núria, Pedrós Barnils^1^, Tilman Brand^2^, Hajo Zeeb^2,3^, Benjamin Schüz^1,3^

^1^Institute of Public Health and Nursing Research, University of Bremen, Bremen, Germany

^2^Department of Prevention and Evaluation, Leibniz Institute for Prevention Research and Epidemiology – BIPS, Bremen, Germany

^3^Health Sciences Bremen, University of Bremen, Bremen, Germany

(1-86)

1. Amoran OE, Fatugase KO, Fatugase OM, Alausa KO. Impact of health education intervention on insecticide treated nets uptake among nursing mothers in rural communities in Nigeria. BMC Research Notes. 2012;5:444.

2. Au LE, Whaley SE, Gurzo K, Meza M, Rosen NJ, Ritchie LD. Evaluation of Online and In-Person Nutrition Education Related to Salt Knowledge and Behaviors among Special Supplemental Nutrition Program for Women, Infants, and Children Participants. Journal of the Academy of Nutrition and Dietetics. 2017;117(9):1384-95.

3. Backman D, Scruggs V, Atiedu AV, Bowie S, Bye L, Dennis A, et al. Using a Toolbox of Tailored Educational Lessons to Improve Fruit, Vegetable, and Physical Activity Behaviors among African American Women in California. Journal of Nutrition Education & Behavior. 2011;43(4):75-85.

4. Bahromov M, Weine S. HIV prevention for migrants in transit: Developing testing train. AIDS Education and Prevention. 2011;23(3):267-80.

5. Berry D, Colindres M, Sanchez-Lugo L, Sanchez M, Neal M, Smith-Miller C. Adapting, Feasibility Testing, and Pilot Testing a Weight Management Intervention for Recently Immigrated Spanish-Speaking Women and Their 2- to 4-Year-Old Children. Hispanic Health Care International (Springer Publishing Company, Inc). 2011;9(4):186-93.

6. Bessems K, Linssen E, Lomme M, van Assema P. The Effectiveness of the Good Affordable Food Intervention for Adults with Low Socioeconomic Status and Small Incomes. Int J Environ Res Public Health. 2020;17(7):2535.

7. Bogale GW, Boer H, Seydel ER. Effects of a theory-based audio HIV/AIDS intervention for illiterate rural females in Amhara, Ethiopia. AIDS Educ Prev. 2011;23(1):25-37.

8. Bucher HC, Eser T, Weinbacher M, Gyr K. HIV prevention in Turkish immigrants in a general internal medicine outpatient clinic. Sozial- und Praventivmedizin. 1997;42(1):21-9.

9. Cala Cala LF, Kelly CL, Ramos E, VanVleet M, High P. Which Mothers Know That All Babies Cry? A Randomized Controlled Trial of a Child Abuse Prevention Program for Low-Income New Mothers. Clinical Pediatrics. 2020;59(9):865-73.

10. Calderón-Mora J, Byrd TL, Alomari A, Salaiz R, Dwivedi A, Mallawaarachchi I, et al. Group Versus Individual Culturally Tailored and Theory-Based Education to Promote Cervical Cancer Screening Among the Underserved Hispanics: A Cluster Randomized Trial. Am J Health Promot. 2020;34(1):15-24.

11. Campbell MK, Carbone E, Honess-Morreale L, Heisler-MacKinnon J, Demissie S, Farrell D. Randomized Trial of a Tailored Nutrition Education CD-ROM Program for Women Receiving Food Assistance. Journal of Nutrition Education and Behavior. 2004;36(2):58-66.

12. Carey MP, Braaten LS, Maisto SA, Gleason JR, Forsyth AD, Durant LE, et al. Using information, motivational enhancement, and skills training to reduce the risk of HIV infection for low-income urban women: a second randomized clinical trial. Health Psychol. 2000;19(1):3-11.

13. Chen W, Li T, Zou G, Renzaho AMN, Li X, Shi L, et al. Results of a Cluster Randomized Controlled Trial to Promote the Use of Respiratory Protective Equipment among Migrant Workers Exposed to Organic Solvents in Small and Medium-Sized Enterprises. Int J Environ Res Public Health. 2019;16(17):3187.

14. Choi SY. Development of an educational program to prevent cervical cancer among immigrants in Korea. Asian Pacific Journal of Cancer Prevention. 2013;14(9):5345-9.

15. Choi YJ. Effects of a Program to Improve Mental Health Literacy for Married Immigrant Women in Korea. Archives of Psychiatric Nursing. 2017;31(4):394-8.

16. Cianelli R, Ferrer L, Norr KF, Miner S, Irarrazabal L, Bernales M, et al. Mano a Mano-Mujer: An Effective HIV Prevention Intervention for Chilean Women. Health Care for Women International. 2012;33(4):321-41.

17. Dancy BL, Marcantonio R, Norr K. The long-term effectiveness of an HIV prevention intervention for low-income African American women. AIDS Educ Prev. 2000;12(2):113-25.

18. Fitzgibbon ML, Stolley MR, Avellone ME, Sugerman S, Chavez N. Involving Parents in Cancer Risk Reduction: A Program for Hispanic American Families. Health Psychology. 1996;15(6):413-22.

19. Flaskerud JH, Nyamathi AM. Effects of an AIDS education program on the knowledge, attitudes and practices of low income black and Latina women. J Community Health. 1990;15(6):343-55.

20. Frosch DL, Uy V, Ochoa S, Mangione CM. Evaluation of a behavior support intervention for patients with poorly controlled diabetes. Arch Intern Med. 2011;171(22):2011-7.

21. García A, Brown SA, Horner SD, Zuñiga J, Arheart KL. Home-based diabetes symptom self-management education for Mexican Americans with type 2 diabetes. Health Education Research. 2015;30(3):484-96.

22. Gerber BS, Brodsky IG, Lawless KA, Smolin LI, Arozullah AM, Smith EV, et al. Implementation and evaluation of a low-literacy diabetes education computer multimedia application. Diabetes Care. 2005;28(7):1574-80.

23. Ghahremani L, Azizi M, Moemenbellah-Fard MD, Ghaem H. Malaria preventive behaviors among housewives in suburbs of Bandar-Abbas City, south of Iran: interventional design based on PRECEDE model. Pathogens and Global Health. 2019;113(1):32-8.

24. Gittelsohn J, Song HJ, Suratkar S, Kumar MB, Henry EG, Sharma S, et al. An Urban Food Store Intervention Positively Affects Food-Related Psychosocial Variables and Food Behaviors. Health Education & Behavior. 2010;37(3):390-402.

25. Gollub EL, Morrow KM, Mayer KH, Koblin BA, Peterside PB, Husnik MJ, et al. Three city feasibility study of a body empowerment and HIV prevention intervention among women with drug use histories: Women FIT. Journal of Women's Health. 2010;19(9):1705-13.

26. Groessl EJ, Ho SB, Asch SM, Stepnowsky CJ, Laurent D, Gifford AL. The Hepatitis C Self-Management Program: Sustainability of Primary Outcomes at 1 Year. Health Education and Behavior. 2013;40(6):730-40.

27. Havas S, Anliker J, Greenberg D, Block G, Block T, Blik C, et al. Final results of the Maryland WIC Food for Life Program. Prev Med. 2003;37(5):406-16.

28. Heisler M, Choi H, Palmisano G, Mase R, Richardson C, Fagerlin A, et al. Comparison of community health worker-led diabetes medication decision-making support for low-income latino and african american adults with diabetes using E-health tools versus print materials. Annals of Internal Medicine. 2014;161:13-22.

29. Henshaw MM, Borrelli B, Gregorich SE, Heaton B, Tooley EM, Santo W, et al. Randomized Trial of Motivational Interviewing to Prevent Early Childhood Caries in Public Housing. JDR Clinical and Translational Research. 2018;3(4):353-65.

30. Hernandez MY, Organista KC. Entertainment-Education? A Fotonovela? A New Strategy to Improve Depression Literacy and Help-Seeking Behaviors in At-Risk Immigrant Latinas. American Journal of Community Psychology. 2013;52(3):224-35.

31. Hidiroglu S, Topuzoglu A, Onsuz MF. A community-based intervention programme on hormonal contraceptives: The utilisation of social networks. Journal of Obstetrics and Gynaecology. 2016;36(1):126-30.

32. Hoddinott J, Ahmed A, Karachiwalla NI, Roy S. Nutrition behaviour change communication causes sustained effects on IYCN knowledge in two cluster-randomised trials in Bangladesh. Maternal and Child Nutrition. 2018;14(1):e12498.

33. Hoque BA, Juncker T, Sack RB, Ali M, Aziz KMA. Sustainability of a water, sanitation and hygiene education project in rural Bangladesh: A 5-year follow-up. Bulletin of the World Health Organization. 1996;74(4):431-7.

34. Horodynski MA, Stommel M. Nutrition education aimed at toddlers: an intervention study. Pediatr Nurs. 2005;31(5):364, 7-72.

35. Howard-Pitney B, Winkleby MA, Albright CL, Bruce B, Fortmann SP. The Stanford Nutrition Action Program: A dietary fat intervention for low-literacy adults. Am J Public Health. 1997;87(12):1971-6.

36. Hughes SC, Corcos I, Hovell M, Hofstetter CR. Feasibility Pilot of a Randomized Faith-Based Intervention to Reduce Secondhand Smoke Exposure Among Korean Americans. Preventing chronic disease. 2017;14:E19.

37. Hughes SC, Obayashi S. Faith-based intervention to increase fruit and vegetable intake among Koreans in the USA: a feasibility pilot. Public Health Nutrition. 2016;20(2):357-62.

38. Hughes SO, Power TG, Beck A, Betz D, Goodell LS, Hopwood V, et al. Short-Term Effects of an Obesity Prevention Program Among Low-Income Hispanic Families With Preschoolers. Journal of Nutrition Education and Behavior. 2020;52(3):224-39.

39. Jay M, Adams J, Herring SJ, Gillespie C, Ark T, Feldman H, et al. A randomized trial of a brief multimedia intervention to improve comprehension of food labels. Preventive Medicine. 2008;48(1):25-31.

40. Jervelund SS, Maltesen T, Wimmelmann CL, Petersen JH, Krasnik A. Ignorance is not bliss: The effect of systematic information on immigrants' knowledge of and satisfaction with the Danish healthcare system. Scand J Public Health. 2017;45(2):161-74.

41. Jibaja ML, Kingery P, Neff NE, Smith Q, Bowman J, Holcomb JD. Tailored, interactive soap operas for breast cancer education of high-risk Hispanic women. Journal of Cancer Education. 2000;15(4):237-42.

42. Kaur N, elman D, Potvin L. Effectiveness of Safeguard your smile,” an oral health literacy intervention, on oral hygiene self-care behaviour among Punjabi immigrants: A randomized controlled trial. Canadian Journal of Dental Hygiene. 2019;53(1):23-32.

43. Kendall P, Scharff R, Baker S, LeJeune J, Sofos J, Medeiros L. Food Safety Instruction Improves Knowledge and Behavior Risk and Protection Factors for Foodborne Illnesses in Pregnant Populations. Maternal and Child Health Journal. 2017;21(8):1686-98.

44. Kersten HB, Moughan B, Moran MM, Spector ND, Smals LE, DeLago CW. A videotape to improve parental knowledge of lead poisoning. Ambulatory Pediatrics. 2004;4(4):344-7.

45. Kim J, Kim N. Effects of Birth Control Empowerment Program for Married Immigrant Vietnamese Women in South Korea. Korean Journal of Women Health Nursing. 2017;23:1.

46. Kim JH, Menon U, Wang E, Szalacha L. Assess the Effects of Culturally Relevant Intervention on Breast Cancer Knowledge, Beliefs, and Mammography Use Among Korean American Women. Journal of Immigrant & Minority Health. 2009;12(4):586-97.

47. Kim SS, Nguyen PH, Tran LM, Sanghvi T, Mahmud Z, Haque MR, et al. Large-Scale Social and Behavior Change Communication Interventions Have Sustained Impacts on Infant and Young Child Feeding Knowledge and Practices: Results of a 2-Year Follow-Up Study in Bangladesh. J Nutr. 2018;148(10):1605-14.

48. Kjøllesdal MKR, Hjellset VT, Bjørge B, Holmboe-Ottesen G, el M. Perceptions of risk factors for diabetes among Norwegian-Pakistani women participating in a culturally adapted intervention. Ethnicity and Health. 2010;16(3):279-97.

49. Lawrence JSS, Wilson TE, Eldridge GD, Brasfield TL, O'Bannon RE. Community-based interventions to reduce low income, African American women's risk of sexually transmitted diseases: A randomized controlled trial of three theoretical models. American Journal of Community Psychology. 2001;29(6):937-64.

50. Lee FH, Wang HH, Yang YM, Tsai HM, Huang JJ. The Effects of an Educational Intervention on Preventing Cervical Cancer Among Vietnamese Women in Southern Taiwan. Journal of Cancer Education. 2016;32(3):622-8.

51. Lee P-I, Lai HR, Lin PC, Kuo SY, Lin YK, Chen SR, et al. Effects of a parenting sexual education program for immigrant parents: A cluster randomized trial. Patient Education and Counseling. 2019;103(2):343-9.

52. Li N, Li X, Wang X, Shao J, Dou J. A cross-site intervention in Chinese rural migrants enhances HIV/AIDS knowledge, attitude and behavior. International Journal of Environmental Research and Public Health. 2014;11(4):4528-43.

53. Li X, Lin D, Wang B, Du H, Tain CC, Stanton B. Efficacy of theory-based hiv behavioral prevention among rural-to-urban migrants in china: A randomized controlled trial. AIDS Education and Prevention. 2014;26(4):296-316.

54. LoRe D, Leung CYY, Brenner L, Suskind DL. Parent-directed intervention in promoting knowledge of pediatric nutrition and healthy lifestyle among low-SES families with toddlers: A randomized controlled trial. Child: Care, Health and Development. 2019;45(4):518-22.

55. Luque JS, Tarasenko YN, Reyes-Garcia C, Alfonso ML, Suazo N, Rebing L, et al. Salud es Vida: a Cervical Cancer Screening Intervention for Rural Latina Immigrant Women. Journal of Cancer Education. 2016;32(4):690-9.

56. Monterrosa EC, Frongillo EA, de Cossío TG, Bonvecchio A, Villanueva MA, Thrasher JF, et al. Scripted messages delivered by nurses and radio changed beliefs, attitudes, intentions, and behaviors regarding infant and young child feeding in mexico. Journal of Nutrition. 2013;143(6):915-22.

57. Navarro AM, Raman R, McNicholas LJ, Loza O. Diffusion of cancer education information through a Latino community health advisor program. Preventive Medicine. 2007;45(2):135-8.

58. Nguyen PH, Frongillo EA, Sanghvi T, Wable G, Mahmud Z, Tran LM, et al. Engagement of Husbands in a Maternal Nutrition Program Substantially Contributed to Greater Intake of Micronutrient Supplements and Dietary Diversity during Pregnancy: Results of a Cluster-Randomized Program Evaluation in Bangladesh. J Nutr. 2018;148(8):1352-63.

59. Nyamathi A, Stein JA, Schumann A, Tyler D. Latent variable assessment of outcomes in a nurse-managed intervention to increase latent tuberculosis treatment completion in homeless adults. Health Psychol. 2007;26(1):68-76.

60. Nyamathi AM, Kington RS, Flaskerud J, Lewis C, Leake B, Gelberg L. Two-Year Follow-Up of AIDS Education Programs for Impoverished Women. Western Journal of Nursing Research. 1999;21(3):405-25.

61. Nyamathi AM, Leake B, Flaskerud J, Lewis C, Bennett C. Outcomes of specialized and traditional AIDS counseling programs for impoverished women of color. Res Nurs Health. 1993;16(1):11-21.

62. Odom SE. Effects of an educational intervention on mothers of male children with attention deficit hyperactivity disorder. J Community Health Nurs. 1996;13(4):207-20.

63. Orhan C, Lenoir D, Favoreel A, Van Looveren E, Yildiz Kabak V, Mukhtar NB, et al. Culture-sensitive and standard pain neuroscience education improves pain, disability, and pain cognitions in first-generation Turkish migrants with chronic low back pain: a pilot randomized controlled trial. Physiother Theory Pract. 2021;37(5):633-45.

64. Patel MR, Israel BA, Song PXK, Hao W, TerHaar L, Tariq M, et al. Insuring Good Health: Outcomes and Acceptability of a Participatory Health Insurance Literacy Intervention in Diverse Urban Communities. Health Education and Behavior. 2019;46(3):494-505.

65. Pokharel P, Shettigar PG. Impact of counseling in knowledge, attitude and practice and association of nutritional status with CD4 count and opportunistic infections of HIV patients of Udupi, India. Clinical Nutrition ESPEN. 2019;29:154-9.

66. Rahman A, Iqbal Z, Roberts C, Husain N. Cluster randomized trial of a parent-based intervention to support early development of children in a low-income country. Child: Care, Health and Development. 2009;35(1):56-62.

67. Ratnapradipa D, Quilliam D, Wier L, Rhodes DL. Food safety education: Child-to-parent instruction in an immigrant population. Journal of Environmental Health. 2011;73(6):70-5.

68. Reznik M, Ozuah PO. Asthma educational videoconferencing for parents: A case-control study. Journal of Telemedicine and Telecare. 2004;10:83-5.

69. Sánchez JP, Kaltwassar S, McClellan M, Burton WB, Blank A, Calderon Y. Educational video tool to increase syphilis knowledge among black and Hispanic male patients. J Health Care Poor Underserved. 2010;21(1):371-85.

70. Sanderson M, Khabele D, Brown CL, Harbi K, Alexander LR, Coker AL, et al. Results of a Health Education Message Intervention on HPV Knowledge and Receipt of Follow-up Care among Latinas Infected with High-risk Human Papillomavirus. J Health Care Poor Underserved. 2015;26(4):1440-55.

71. Scheinmann R, Chiasson MA, Hartel D, Rosenberg TJ. Evaluating a bilingual video to improve infant feeding knowledge and behavior among immigrant latina mothers. Journal of Community Health. 2009;35(5):464-70.

72. Schwebel DC, Swart D, Simpson J, Hui SKA, Hobe P. An Intervention to Reduce Kerosene-Related Burns and Poisonings in Low-Income South African Communities. Health Psychology. 2009;28(4):493-500.

73. Singh A, Klemm RDW, Mundy G, ey Rana P, Pun B, Cunningham K. Improving maternal, infant and young child nutrition in Nepal via peer mobilization. Public Health Nutrition. 2017;21(4):796-806.

74. Soto Mas F, Jacobson HE, Olivárez A. Adult Education and the Health Literacy of Hispanic Immigrants in the United States. Journal of Latinos and Education. 2017;16(4):314-22.

75. Soto Mas F, Ji M, Fuentes BO, Tinajero J. The health literacy and ESL study: A community-based intervention for spanish-speaking adults. Journal of Health Communication. 2015;20(4):369-76.

76. Soto Mas F, Schmitt CL, Jacobson H, Myers OB. A Cardiovascular Health Intervention for Spanish Speakers: The Health Literacy and ESL Curriculum. Journal of Community Health. 2018;43(4):717-24.

77. Tessaro I, Rye S, Parker L, Mangone C, McCrone S. Effectiveness of a nutrition intervention with rural low-income women. American Journal of Health Behavior. 2007;31(1):35-43.

78. Tsai TL, Lee SYD, Yu WR. Impact of a Problem-Based Learning (PBL) Health Literacy Program on Immigrant Women's Health Literacy, Health Empowerment, Navigation Efficacy, and Health Care Utilization. Journal of Health Communication. 2018;23(4):340-9.

79. Tyler D, Nyamathi A, Stein JA, Koniak-Griffin D, Hodge F, Gelberg L. Increasing hepatitis C knowledge among homeless adults: Results of a community-based, interdisciplinary intervention. Journal of Behavioral Health Services and Research. 2014;41(1):37-49.

80. Unger JB, Cabassa LJ, Molina GB, Contreras S, Baron M. Evaluation of a fotonovela to increase depression knowledge and reduce stigma among hispanic adults. Journal of Immigrant and Minority Health. 2012;15(2):398-406.

81. Van Servellen G, Carpio F, Lopez M, Garcia-Teague L, Herrera G, Monterrosa F, et al. Program to enhance health literacy and treatment adherence in low-income HIV-infected Latino men and women. AIDS Patient Care & STDs. 2003;17(11):581-94.

82. van Servellen G, Nyamathi A, Carpio F, Pearce D, Garcia-Teague L, Herrera G, et al. Effects of a treatment adherence enhancement program on health literacy, patient-provider relationships, and adherence to HAART among low-income HIV-positive Spanish-speaking Latinos. AIDS Patient Care STDS. 2005;19(11):745-59.

83. Wang HH, Lin ML, Yang YM, Tsai HM, Huang JJ. The effects of group health education on childbearing knowledge, attitude, and behaviour among Southeast Asian immigrant women in Taiwan. Midwifery. 2012;28(6):754-9.

84. Wilson FL, Brown DL, Stephens-Ferris M. Can easy-to-read immunization information increase knowledge in urban low-income mothers? Journal of Pediatric Nursing. 2006;21(1):4-12.

85. Zhang T, Tian X, Ma F, Yang Y, Yu F, Zhao Y, et al. Community Based Promotion on VCT Acceptance among Rural Migrants in Shanghai, China. PLoS ONE. 2013;8(4).

86. Zhu C, Geng Q, Chen L, Yang H, Jiang W. Impact of an educational programme on reproductive health among young migrant female workers in Shenzhen, China: An intervention study. International Journal of Behavioral Medicine. 2014;21(4):710-8.

Table 1: Data extraction table - Systematic review: Intervention strategies to promote health literacy in socioeconomically disadvantaged groups

| First author and year of publication | Country where the study was conducted | Study design | Description of intervention | Delivery mode | Health knowledge outcome | Measurement of health knowledge outcome | Sample size - Intervention vs control group (last F/U) | Health knowledge improvement of intervention vs control group  *(see legend) |
| --- | --- | --- | --- | --- | --- | --- | --- | --- |
| Amoran et al. 2012 | Nigeria | Quasi-experimental | Educational programm | Multiple (Face-to-face, written educational materials) | Insecticide treated nets (ITN) knowledge | KAP survey (knowledge, attitude and practice) | 190 vs 180 | Disease causation: ↑ Mounting ITN: ↑ Treatment of ITN: ↑ |
| Au et al. 2017 | USA | Randomized controlled trial | Salt education class | Face-to-face | Salt-related knowledge: main source of dietary salt, amount of sodium adults should consume daily | Self-developed based on class content | 324 & 257 | Main source of dietary salt: 0 Amount of sodium adults should consume daily: 0 |
| Backman et al. 2011 | USA | Quasi-experimental | Toolbox classes covering nutrition and physical activity | Face-to-face | Fruit, vegetable, physical activity-related knowledge | Self-developed | 156 vs 171 | Recommended cups of fruit and vegetables: 0 The number of cups equivalent to a handful of fruits and vegetables: one-half cup: ↑ Three factors determining the amount of fruit and vegetables recommended for individual daily consumption: ↑ (Further tests in original article) |
| Bahromov et al. 2011 | Tajikistan | Cluster randomized controlled trial | Education classes (didactic presentation, role-playing, group discussions) | Face-to-face | HIV & condom use knowledge | Questionnaires adapted from previous surveys | 30 vs 30 | HIV/AIDS knowledge: ↑ Condom use knowledge: ↑ |
| Berry et al. 2011 | USA | Cluster randomized controlled trial | Nutrition and exercise education, coping skills training, physical activity classes (zumba, walking, kickboxing) | Face-to-face | Nutrition and exercise knowledge | Health Promoting Lifestyle Profile II Questionnaire | n=56 in total (both groups) | Exercise knowledge: ↑  Nutrition knowledge: ↑ |
| Bessems et al. 2020 | Netherlands | Quasi-experimental | Group education classes | Face-to-face | Perceived procedural knowledge | Self-developed | 80 vs 72 | Perceived procedural knowledge: ↑ |
| Bogale et al. 2011 | Ethiopia | Quasi-experimental | Audios on HIV prevention in the form of radio programs | Audio | HIV knowledge | Self-developed true/false questionnaire | 210 vs 207 | HIV/AIDS knowledge: ↑ |
| Bucher et al. 1997 | Switzerland | Non-randomized controlled trial | HIV consultation with dolmetschers | Face-to-face | HIV infection knowledge | Based on HIV knowledge questions developed by the Federal Office of Public Health (Germany) | 66 vs 54 | Infection knowledge: 0 |
| Cala Cala et al. 2020 | USA | Randomized controlled trial | Bilingual DVD and bilingual booklets with strategies to soothe parents and their children | Multiple (DVD and written educational materials) | Peak of crying knowledge;  Shaken baby syndrome knowledge | Self-developed | 53 vs 62 | Peak of crying knowledge: ↑  Shaken baby syndrome knowledge: 0 |
| Calderón-Mora et al. 2020 | USA | Cluster randomized controlled trial | Culturally tailored cervical cancer screening education (flipchart, message card, action plan worksheet, resource sheet, handouts) | Multiple (Face-to-face, written educational materials) | Cervical cancer and screening knowledge | Self-developed true/false | 150 vs 150 | A lack of hygiene can cause cervical cancer: 0 If women have regular tests, advanced cervical cancer unlikely: 0  (Further tests in original article) |
| Campbell et al. 2004 | USA | Randomized controlled trial | Interactive CD-ROM including video soap opera, infomercials, dietary assessment, and individually tailored dietary feedback and strategies for change | Multiple (Video, face-to-face and written educational materials) | Infant feeding knowledge  Low fat knowledge | Self-developed | 307 | Infant feeding knowledge: ↑  Low fat knowledge: ↑ |
| Carey et al. 2000 | USA | Randomized controlled trial | HIV education by using motivational interviewing | Face-to-face | HIV knowledge | HIV Knowledge Questionnaire (HIV-K-Q) | 43 vs 34 | Mean HIV knowledge: ↑ |
| Chen et al. 2019 | China | Cluster randomized controlled trial | Passive occupational health education and training module and mHealth, peer education component | Multiple (Illustrative pictures, videos, face-to-face, leaflets, posters) | Occupational health knowledge | Self-developed | 324 vs 332 | Occupational health knowledge: ↑ |
| Choi et al. 2013 | South Korea | Non-randomized controlled trial | Cervical cancer prevention education | Multiple (face-to-face, picture book) | HPV knowledge | Validated HPV questionnaire | 30 vs 27 | Cervical cancer knowledge: ↑ |
| Choi et al. 2017 | South Korea | Non-randomized controlled trial | Provision of mental health services based on a mental health guidebook comprising 8 sections | Face-to-face | Mental health literacy | Mental health Literacy Inventory | 31 vs 32 | Mental health literacy: ↑ |
| Cianelli et al. 2012 | Chile | Quasi-experimental | HIV education guided by a conceptual framework integrating the social-cognitive model of behavioral change, contextual tailoring, and the World Health Organization’s primary health care model | Face-to-face | HIV knowledge | 12-item scale created by Heckman et al. | 182 vs 218 | HIV Knowledge: ↑ |
| Dancy et al. 2000 | USA | Non-randomized controlled trial | Experiential training in HIV risk reduction strategies | Face-to-face | Condom knowledge | Self-developed health questionnaire | 98 vs 98 | Condom knowledge: 0 |
| Fitzgibbon et al. 1996 | USA | Randomized controlled trial | Culture specific cancer education classes | Face-to-face | Mothers' and children's nutrition knowledge | Nutrition Knowledge Questionnaire | 18 vs 18 | Nutrition knowledge: 0 |
| Flaskerud et al. 1990 | USA | Non-randomized controlled trial | AIDS education (slide-tape, brochure) | Written educational materials | AIDS knowledge | Self-developed | 406 vs 206 | AIDS knowledge (total score): ↑ |
| Frosch et al. 2011 | USA | Randomized controlled trial | Diabetes DVD program with booklet | Multiple (DVD and telephone) | Diabetes knowledge | Diabetes Knowledge Test, developed by the University of Michigan Diabetes Research and Training Center. | 95 vs 87 | Diabetes knowledge: 0 |
| García et al. 2015 | USA | Randomized controlled trial | In-home, educational and sessions with a registered nurse followed by eight biweekly support telephone sessions | Face-to-face | Diabetes knowledge | The Spoken Knowledge in Low Literacy in Diabetes (SKILLD) Scale | 28 vs 24 | Diabetes knowledge: 0 |
| Gerber et al. 2005 | USA | Randomized controlled trial | Computer-based multimedia lessons | Online | Diabetes knowledge | Self-developed | 122 vs 122 | Diabetes knowledge: 0 |
| Ghahremani et al. 2019 | Iran | Quasi-experimental | Educational program | Face-to-face | Malaria preventive behavior knowledge | Self-developed | 86 vs 86 | Malaria preventive behavior knowledge: ↑ |
| Gittelsohn et al. 2010 | USA | Quasi-experimental | Changing local food environment, providing healthier food options | Face-to-face | Food knowledge | Customer Impact Questionnaire | 45 vs 39 | Food knowledge: 0 |
| Gollub et al. 2010 | USA | Quasi-experimental | HIV education, contextualization of skills from education session, focusing on women´s bodies and health needs | Face-to-face | Body knowledge; prevention methods knowledge | Self-developed | 91 vs 98 (baseline. F/U not reported) | Body knowledge: ↑  Prevention methods knowledge: ↑ |
| Groessl et al. 2013 | USA | Randomized controlled trial | HCV self-management program and educational materials (booklet, book) | Multiple (face-to-face and written education materials) | HCV knowledge | Self-developed | 52 vs 45 | HCV knowledge: ↑ |
| Havas et al. 2003 | USA | Cluster randomized controlled trial | Video, brochure, workshops, newsletters, mail packets, behavior-enforcing incentives, phone calls | Multiple (face-to-face, online, telephone) | Food knowledge | Questionnaire incorporating a food list based on dietary data from the Third National Health and Nutrition Examination Survey (NHANES III) | 753 vs 755 | Knowledge fat: ↑  Knowledge fruit & vegetables: ↑  Knowledge fiber: ↑ |
| Heisler et al. 2014 | USA | Randomized controlled trial | One-on-one education, printed materials | Multiple (face-to-face, written educational materials, online) | Anti-hyperglemic medication knowledge | Self-developed | 87 vs 89 | Anti-hyperglycemic medication knowledge: 0 |
| Henshaw et al. 2018 | USA | Cluster randomized controlled trial | Provision of toothbrush, quarterly counseling, written handouts | Mutiple (face-to-face, written educational materials) | Oral health knowledge | Self-developed | 297 vs 582 | Oral health knowledge: ↑ |
| Hernandez et al. 2013 | USA | Quasi-experimental | Fotonovela presenting middle-aged depressed Latina | Face-to-face with fotonovelas | Depression knowledge | Developed by Unger et al. 2012 | 75 vs 67 | Depression knowledge: ↑ |
| Hidiroglu et al. 2015 | Turkey | Quasi-experimental | Training sessions conducted within an organized festival | Face-to-face | Contraceptive knowledge | Self-developed | 366 vs 437 | Ever heard of emergency contraception: ↑ Knowing at least one advantage of oral contraceptives: ↑ |
| Hoddinott et al. 2018 | Bangladesh | Cluster randomized controlled trial | Two IGs - a) cash transfer, b) food ration | Face-to-face | Breastfeeding knowledge | Self-developed | 2341 (in total) | Breastfeeding knowledge: ↑ |
| Hoque et al. 1996 | Bangladesh | Cluster randomized controlled trial | Provision of handpumps, hygiene education on hygiene practices | Face-to-face group sessions | Transmission of diarrhoea knowledge | Knowledge about diarrhoea study questionnaire | 617 vs 451 | Knowledge about transmission of diarrhoea: ↑ |
| Horodynski et al. 2005 | USA | Quasi-experimental | Group-based nutrition lessons, individually structured reinforcement activities (videotapes, hands-on learning activities) | Multiple (face-to-face, video) | Toddler feeding knowledge | Self-developed 16 facts sheet | 62 & 43 | Toddler feeding knowledge: ↑ |
| Howard-Pitney et al. 1997 | USA | Quasi-experimental | Culturally appropriate classroom-based and maintenance intervention (nutrition classes, interactive discussion, skill-building tasks, food tastings, demonstrations) | Multiple (face-to-face and written educational materials) | Nutrition knowledge | Self-developed true/false | 183 vs 168 | Nutrition knowledge: ↑ |
| Hughes et al. 2017 | USA | Cluster randomized controlled trial | Motivational interviewing by telephone, binder with information about second hand smoke constituents | Mutiple (telephone, written educational materials) | Second hand smoke knowledge | Self-developed | 40 vs 35 | Secondhand smoke knowledge: ↑ |
| Hughes et al. 2016 | USA | Cluster randomized controlled trial | Education materials (binder with exercises to identify important values related to healthy eating, benefits of fruits and vegetables), coaching sessions, church activities | Multiple (telephone, face-to-face) | Nutrition knowledge | Feed knowledge questionnaire | 35 vs 36 | Most fresh fruits & vegetables have almost no sodium: ↑  Recommended intake for fruits: ↑  Recommended intake for vegetables: ↑ |
| Hughes et al. 2020 | USA | Randomized controlled trial | Multicomponent family-based prevention program, parent-child education sessions | Face-to-face | Feeding Knowledge | Feeding Knowledge Questionnaire | 101 vs 77 | Best practices: ↑ Misconceptions: ↑ Child roles: ↑  (Further tests in original article) |
| Jay et al. 2008 | USA | Randomized controlled trial | Interactive question-answer video educating about healthy foods in a supermarket | Video | Nutrition knowledge | Self-developed | 23 vs 19 | Nutrition knowledge: ↑ |
| Jeverlund et al. 2017 | Denmark | Quasi-exprimental | Two IGs - a) information material (booklet-translated in 9 languages) b) booklet + course with exercises and empirical examples that encouraged discussion, visit by GP to discuss expectations and roles of patients | Multiple (face-to-face, written materials) | Danish health care system knowledge | Self-developed | 307 vs 364 vs 373 | Self-perceived knowledge in event of an accident: ↑ |
| Jibaja et al. 2000 | USA | Randomized controlled trial | Computer-tailored program with combination of pictures and video | Online | Breast cancer screening knowledge and beliefs | Self-developed | 118 vs 60 | Breast cancer knowledge/beliefs score: 0 |
| Kaur et al. 2019 | Canada | Randomized controlled trial | Fotonovela, demonstration of adequate tools and skills of toothbrushing, encouragement to track oral hygiene process | Multiple (face-to-face, telephone, fotonovelas) | Oral health literacy (OHL) and oral hygiene self-care knowledge | Rapid Estimate of Adult Literacy in Dentistry (TS-REALD) | 70 vs 70 | Oral health literacy: ↑ |
| Kendall et al. 2017 | USA | Randomized controlled trial | 8-lesson curriculum (education lessons, home visits) | Face-to-face | Food safety knowledge | Self-developed | 312 | Multiple outcomes – see original article |
| Kersten et al. 2004 | USA | Non-randomized controlled trial | Education videotape | Video | Lead Poisoning Knowledge | Shortened Chicago Lead Knowledge Test (sCLKT) with 14 questions. sCLKT score created based on number of correct answers- range 0-14 | 73 & 35 | Lead posioning knowledge: ↑ |
| Kim et al. 2018 | Bangladesh | Cluster randomized controlled trial | Standard nutrition counseling, TV spots with messages | Multiple (face-to-face, TV spots) | Breastfeeding (BF) and complementary feeding (CF) knowledge | Self-developed | 1095 & 1001 & 1200 | BF knowledge: ↑  CF knowledge: 0 |
| Kim et al. 2017 | South Korea | Randomized controlled trial | Information sessions, group discussions | Multiple (face-to-face, written materials) | Contraceptive knowledge | Birth Control Knowledge Measuring Scale | 23 vs 23 | Contraceptive knowledge: ↑ |
| Kim et al. 2009 | USA | Quasi-experimental | Education program with powerpoint slides | Face-to-face | Mammogram screening knowledge | the Champion Breast Cancer Survey | 90 vs 90 | Mammogram screening knowledge: ↑ |
| Kjøllesdal et al. 2010 | Norway | Randomized controlled trial | Group sessions with culturally adapted and translated materials | Face-to-face | Food knowledge (food perceptions) | Self-developed questions on which foods are healthy and which ones are unhealthy | 101 vs 97 (baseline) | Food knowledge: ↑ |
| Lawrence et al. 2001 | USA | Quasi-experimental | Information in form of videotapes telling HIV stories, skill observation | Multiple (face-to-face, videotapes) | AIDS Knowledge | Self-developed | 445 | AIDS knowledge: ↑ |
| Lee et al. 2016 | Taiwan | Quasi-experimental | Health education brochure, telephone counseling | Written educational materials, telephone | Cervical cancer knowledge | Modified Knowledge of Cervical Cancer Scale (KCCS) | 112 vs 112 | Cervical cancer knowledge: ↑ |
| Lee et al. 2019 | Taiwan | Cluster randomized controlled trial | Booklet and booster session (including lectures, discussions, watching videos, and role-playing practice) | Multiple (face-to-face, video, written educational materials) | Sexual knowledge | Self-developed | 65 vs 67 | Sexual knowledge: ↑ |
| Li et al. 2014 | China | Randomized controlled trial | Behavioral intervention sessions (multimedia presentations, discussions, role-play, group exercises, games) | Multiple (face-to-face, video, written educational materials) | Condom Use Knowledge, HIV/AIDS Knowledge | Self-developed | 263 vs 216 | Condom use knowledge: ↑ |
| Li et al. 2014 | China | Quasi-experimental | Behavioral intervention sessions (including multimedia presentations, discussions, role-play, group exercise and games) | Multiple (face-to-face, video, written educational materials) | HIV/AIDS knowledge | Self-developed | 266 vs 263 | HIV knowledge: ↑ |
| Lore et al. 2019 | USA | Quasi-experimental | Computer-based curriculum in one-on-one educational sessions | Face-to-face computer-based curriculum | Physical activity and paedetrical nutrition knowledge | Self-developed | 55 vs 49 | Total knowledge score: ↑ |
| Luque et al. 2016 | USA | Quasi-experimental | Group sessions (brochures, flipchart, animated video, in-class activities) | Multiple (face-to-face, video, written educational materials) | Cervical cancer knowledge | Self-developed | 38 vs 52 | Cervical cancer knowledge: ↑ |
|  |  |  |  |  |  |  |  |  |
| Monterrosa et al. 2012 | Mexico | Quasi-experimental | Focus group discussions, scripted messages by nurses on radio | Face-to-face, radio | Child feeding behaviors knowledge | Self-developed | 239 vs 186 | Vegetables keep my baby well nourished: ↓ Beef prevents anemia: ↑ Chicken prevents anemia: ↑  (Further tests in original article) |
| Navarro et al. 2007 | USA | Non-randomized controlled trial | Interactive educational group sessions | Face-to-face | Cervical cancer screening knowledge | Self-developed | 285 vs 222 | Knows mammography recommendation for her age group: ↑  Knows breast self-examination: ↑   (Further tests in original article) |
| Nguyen et al. 2018 | Bangladesh | Cluster randomized controlled trial | Nutrition focused program (interpersonal counseling, distribution of free micronutrient supplements, home visits for pregnant women) | Face-to-face | Food (husbands) knowledge | Self-developed | 622 vs 685 | Total food knowledge score: ↑ |
| Nyamathi et al. 1999 | USA | Quasi-experimental | Culturally component program of AIDS education | Face-to-face | AIDS knowledge | Self-developed | 201 vs 209 | AIDS knowledge: ↓ (traditional group scoring better) |
| Nyamathi et al. 2007 | USA | Quasi-experimental | Educational skills traning module (i.a. Tubercolosis and HIV risk education, training in coping, self-management and communication skills, training in social and problem solving | Face-to-face | Tubercolosis knowledge | Self-developed | 494 (in total) | Tubercolosis knowledge: ↑ |
| Nyamathi et al. 1993 | USA | Quasi-experimental | AIDS videotape, culturally sensitive AIDS education, HIV antibody counseling | Face-to-face with written educational materials | AIDS knowledge and attitudes | Self-developed | 448 vs 410 | AIDS knowledge: ↓ (traditional group scoring better) |
| Odom et al. 1996 | USA | Randomized controlled trial | ADHD educational program | Face-to-face | ADHD knowledge | The ADHD Knowledge and Opinion scale (AKOS) | 10 vs 10 | ADHD overall knowledge: ↑ |
| Orhan et al. 2019 | Belgium | Randomized controlled trial | Pain neuroscience education program in Turkish | Face-to-face with written educational materials | Neurophysiology and chronic low back pain knowledge | Self-developed | 11 vs 10 | Knowledge of pain neurophysiology: 0 |
| Patel et al. 2019 | USA | Cluster randomized controlled trial | Animated videos of superhero characters navigating health care issue | Videos/online | Insurance knowledge | Self-developed | 209 (in total) | Insurance knowledge: specific numbers not reported, IG with stronger beliefs about preventive care than CG |
| Pokharel et al. 2019 | India | Non-randomized controlled trial | Nutrition and health education counseling (leaflet and sample menus) | Face-to-face/telephone | HIV knowledge and practice | Self-developed | 33 & 33 | HIV knowledge: ↑ |
| Rahman et al. 2008 | Pakistan | Cluster randomized controlled trial | Workshop, "Learning Through Play" calendar, homevisits of lady health care workers | Face-to-face and written materials | Child development knowledge | Self-developed | 177 vs 157 | Infant development questionnaire: ↑ |
| Ratnapradipa et al. 2011 | USA | Quasi-experimental | Food handling education by participants children | Face-to-face | Food handling knowledge | Via 13 questions-item randomly selected from the Salt Lake Valley Health Department (SLVHD) testbank | 15 vs 17 | Food handling knowledge: ↑ |
| Reznik et al. 2004 | USA | Non-randomized controlled trial | Asthma education by video conference | Video, online | Asthma knowledge | Self-developed | 47 vs 43 | Asthma knowledge: ↑ |
| Sánchez et al. 2010 | USA | Randomized controlled trial | Video development commenced with a literature review | Video | Syphilis knowledge | Self-developed | 100 vs 106 | Syphilis can be transmitted by oral sex: ↑ Syphilis transmission can be prevented by wearing condoms during sex: ↑  (Further tests in original article) |
| Sanderson et al. 2015 | USA | Quasi-experimental | HPV education brochure (available in English and Spanish) | Written educational materials | HPV knowledge | Self-developed | 185 vs 186 | HPV knowledge: 0 |
| Scheinmann et al. 2009 | USA | Quasi-experimental | Food education through take home DVD | DVD, video | Infant feeding knowledge | Questionnaires adapted from the Bright Futures Nutrition series | 142 vs 129 | A sign that the baby was ready to eat solids: ↑  That solids don’t replace formula/breastmilk: ↑ |
| Schwebel et al. 2009 | USA | Quasi-experimental | Train the trainer model, paraprofessionals deliver education intervention materials to citizens | Face-to-face and written materials | Kerosene-related injury knowledge | Self-developed | 100 vs 106 | Kerosene-related injury knowledge: ↑ |
| Singh et al. 2017 | Nepal | Quasi-experimental | Peer facilitators education program including picture books with messages regarding maternal, infant and young child nutrition practices | Face-to-face | Child nutrition knowledge, maternal nutrition knowledge | Self-developed | 945 & 945 | child nutrition knowledge:  breast milk should be given only 6 months: 0 animal-source foods are good for children : 0 fruits and vegetables are good for children: ↑ |
| Soto Mas et al. 2018 | USA | Randomized controlled trial | Health Literacy and English as a second language Curriculum (within cardiovascular disease education and prevention context) | Face-to-face with written educational materials | Functional health literacy | Test of functional health literacy in adults (TOFHLA) | 77 vs 78 | Functional health literacy: ↑ |
| Soto Mas et al. 2017 | USA | Quasi-experimental | GED curriculum, HL content | Face-to-face | Functional health literacy | Short Test of Functional Health Literacy in Adults (S-TOFHLA) | 61 vs 36 | Functional health literacy: 0 |
| Soto Mas et al. 2015 | USA | Randomized controlled trial | HL & ESL curriculum for spanish speaking adults with low English proficiency | Face-to-face | Functional health literacy | English version of the Test of Functional Health Literacy in Adults (TOFHLA) | 77 vs 78 | Functional health literacy: ↑ |
| Tessaro et al. 2007 | USA | Randomized controlled trial | Online food knowledge education | Online, video | Food knowledge (dietary fat) | 34-item food frequency checklist adapted from an instrument originally validated from NHANES II data and adapted in other studies | 131 vs 131 | Number of fruit and vegetable servings per day: 0  Fat grams intake: 0  (Further tests in original article) |
| Tsai et al. 2018 | Taiwan | Quasi-experimental | Problem-based learning | Face-to-face | Communicative and appraisal health literacy | Self-developed | 63 vs 93 | Communicative and appraisal health literacy: 0 |
| Tyler et al. 2014 | USA | Randomized controlled trial | HIV, HBV education in group sessions | Face-to-face | Hepatitis C virus infection Knowledge | Self-developed | 306 vs 444 | Hepatitis knowledge: ↑ |
| Unger et al. 2012 | USA | Randomized controlled trial | Spanish language fotonovela of depressed middle age latina | Fotonovelas, written educational materials | Depression knowledge | Self-developed | 945 vs 945 | Depression knowledge: ↑ |
| Van Servellen et al. 2003 | USA | Quasi-experimental | Instructional support modular program to build confidence in individuals abilities to follow treatment regimen | Face-to-face | HIV knowledge | Self-developed | 41 vs 40 | HIV knowledge: ↑ |
| Van Servellen et al. 2005 | USA | Randomized controlled trial | Instructional modular program including info-sessions and interactive group exercises | Face-to-face | HIV health literacy | Self-developed | 43 vs 42 | Global HIV disease/treatment knowledge: 0  Recognition HIV terms: 0 |
| Hsiu-Hung Wang et al. 2012 | Taiwan | Quasi-experimental | Maternal health care group health education programme | Multiple (face-to-face, written educational materials, telephone) | Childbearing knowledge | Child bearing knowledge scale | 49 vs 50 | Childbearing knowledge scale: ↑ |
| Wilson et al. 2006 | USA | Randomized controlled trial (pilot) | Easy to read immunization pamphlets | Written educational materials | Immunization knowledge | W-KAIT (knowledge test) | 19 vs 18 | Immunization knowledge: 0 |
| Zhang et al. 2013 | China | Quasi-experimental | Community mobilization and voluntary HIV counseling and testing | Face-to-face with written educational materials | HIV knowledge | Self-developed | 839 vs 434 vs 577 | HIV knowledge: ↑ |
| Zhu et al. 2014 | China | Cluster randomized controlled trial | Reproductive health educational materials, lectures, counseling, access to contraceptives | Face-to-face with written educational materials | Reproductive health knowledge | Self-developed | 1060 vs 2139 | Ever heard of emergency contraception: ↑ Induced abortion is serious (%):↑  AIDS is an STD (%):↑  (Further tests in original article) |

*↑: Health knowledge of intervention group improved statistically significantly compared to the control group

0: Health knowledge of intervention group didn´t change statistically significantly compared to the control group

↓: Health knowledge of control group improved statistically significantly compared to the intervention group

IG: Intervention groups

CG: Control group
