# Supplementary material for: Interventions to promote health literacy among working-age populations experiencing socioeconomic disadvantage: systematic review
Source: Front Public Health. 2024 Feb 19;12:1332720. doi: 10.3389/fpubh.2024.1332720 (PMC10909862; doi:10.3389/fpubh.2024.1332720)
Supplement: Supplementary file 1 [file Data_Sheet_1.ZIP › Supplementary file 3_Albatross_plots and effect sizes.docx]

# Supplementary file 3 – Effect sizes (cohen´s d) of albatross plots

# Interventions to promote health literacy among socioeconomically disadvantaged working-age population groups: Systematic review

Himal Singh^1*^, Florence Samkange-Zeeb^2^, Jonathan Kolschen^1^, Ruben Herrmann^1^, Wiebke Hübner^2^, Núria, Pedrós Barnils^1^, Tilman Brand^2^, Hajo Zeeb^2,3^, Benjamin Schüz^1,3^

^1^Institute of Public Health and Nursing Research, University of Bremen, Bremen, Germany

^2^Department of Prevention and Evaluation, Leibniz Institute for Prevention Research and Epidemiology – BIPS, Bremen, Germany

^3^Health Sciences Bremen, University of Bremen, Bremen, Germany

Table 1: Albatross plot - Functional health literacy

| Author, year of publication | Outcomes | Measurement tool | Sample size | p-value | Effect direction |
| --- | --- | --- | --- | --- | --- |
| Soto mas et al. 2015 | Functional health literacy | TOFHLA | 155 | 0.01 | 0.42 |
| Soto mas et al. 2017 | Functional health literacy | S-TOFHLA | 97 | 0.60 | 0.14 |
| Soto mas et al. 2018 | Functional health literacy | TOFHLA | 155 | 0.012 | 0.41 |
| Tsai et al. 2018 | Communicative and appraisal health literacy | Self-developed | 156 | 0.96 | 0 |

Table 2: Albatross plot - Mental health literacy

| Author, year of publication | Outcomes | Measurement tool | Sample size | p-value | Effect direction |
| --- | --- | --- | --- | --- | --- |
| Choi et al. 2017 | Mental health literacy | MHL Inventory | 63 | 0.001 | 0.72 |
| Hernandez et al. 2013 | Depression knowledge | Developed by Unger et al. 2012^1^ | 142 | 0.001 | 1.19 |
| Odom et al. 1996 | ADHD knowledge | ADHD knowledge and opinion Scale | 20 | 0.09 | 0.78 |
| Unger et al. 2012 | Depression knowledge | Self-developed | 185 | 0.05 | 5.10 |

ADHD: Attention deficit hyperactivity disorder

MHL: Mental health literacy

Table 3: Cancer screening knowledge

| Author, year of publication | Outcomes | Measurement tool | Sample size | p-value | Effect direction |
| --- | --- | --- | --- | --- | --- |
| Calderón-Mora et al. 2020 | A lack of hygiene can cause cervical cancer | Self-developed true/false | 300 | 0.12 | 0.4 |
| Calderón-Mora et al. 2020 | If women have regular tests, advanced cervical cancer unlikely | Self-developed true/false | 300 | 0.96 | 0.01 |
| Calderón-Mora et al. 2020 | A pap test can only detect advanced (invasive) cervical cancer | Self-developed true/false | 300 | 0.15 | 0.27 |
| Calderón-Mora et al. 2020 | I need a pap test only when I experience problems like pain or vaginal bleeding that is not my period | Self-developed true/false | 300 | 0.05 | 1.14 |
| Calderón-Mora et al. 2020 | A pap test is important for a woman under 50 years | Self-developed true/false | 300 | 1 | 0 |
| Calderón-Mora et al. 2020 | Only women who have had many sex partners neet to get a pap test | Self-developed true/false | 300 | 1 | 0 |
| Calderón-Mora et al. 2020 | Pap testing is done through drawing your blood | Self-developed true/false | 300 | 1 | 0 |
| Calderón-Mora et al. 2020 | Women who have gone through menopause still need pap test | Self-developed true/false | 300 | 1 | 0 |
| Jibaja et al. 2000 | Breast cancer screening knowledge and beliefs (younger group: 18-41) | Self-developed true/false | 88 | 0.099 | 0.36 |
| Jibaja et al. 2000 | Breast cancer screening knowledge and belies (older group: 41-65) | Self-developed true/false | 90 | 0.6 | 0.1 |
| Luque et al. 2016 | Cervical cancer screening knowledge | Cervical cancer knowledge instrument | 90 | 0.01 | 0.99 |
| Navarro et al. 2007 | Names at least one breast cancer screening test | Self-developed | 507 | 0.05 | 0.63 |

Table 4: Albatross plot - Child feeding/maternal nutrition knowledge (child-related knowledge)

| Author, year of publication | Outcomes | Measurement tool | Sample size | p-value | Effect direction |
| --- | --- | --- | --- | --- | --- |
| Cala Cala et al. 2020 | Knowledge of peak crying | Self-developed | 115 | 0.03 | 0.43 |
| Cala Cala et al. 2020 | Knowledge of Shaken Baby Syndrome | Self-developed | 115 | 0.09 | 0.32 |
| Cala Cala et al. 2020 | Knowledge that shaking baby may lead to brain damage/death | Self-developed | 115 | 0.10 | 0.32 |
| Campbell et al. 2004 | Infant feeding knowledge - total knowledge score | Self-developed | 307 | 0.06 | 0.20 |
| Fitzigibbon et al. 1996 | Mothers' and children's nutrition knowledge | Nutrition knowledge Questionnaire | 35 | 0.60 | 0.16 |
| Hoddinott et al. 2018 | Total infant and young child nutrition knowledge (north) | Self-developed | 1213 | 0.001 | 0.88 |
| Hoddinott et al. 2018 | Total infant and young child nutrition knowledge (south) | Self-developed | 1128 | 0.001 | 0.94 |
| Hughes et al. 2020 | Feeding Knowledge Questionnaire: Best practices | Feeding knowledge Questionnaire | 255 | 0.001 | 0.97 |
| Hughes et al. 2020 | Feeding Knowledge Questionnaire: Misconceptions | Feeding knowledge Questionnaire | 255 | 0.001 | 0.74 |
| Hughes et al. 2020 | Feeding Knowledge Questionnaire: Child roles | Feeding knowledge Questionnaire | 255 | 0.001 | 0.60 |
| Hughes et al. 2020 | Feeding Knowledge Questionnaire: Repeated presentation of new foods | Feeding knowledge Questionnaire | 255 | 0.001 | 2.90 |
| Hughes et al. 2020 | Feeding Knowledge Questionnaire: Home Feeding efficacy | Feeding knowledge Questionnaire | 255 | 0.001 | 0.45 |
| Kim et al. 2018 | Total Breastfeeding knowledge score | Self-developed | 2400 | 0.01 | 0.26 |
| Lore et al. 2019 | Parental knowledge of paedratic nutrition and healthy lifestlye | Self-developed | 104 | 0.02 | 0.47 |
| Monterrosa et al. 2012 | Vegetables keep my baby well nourished | Self-developed | 425 | 0.001 | 1.48 |
| Monterrosa et al. 2012 | Beef prevents anemia | Self-developed | 425 | 0.001 | 1.50 |
| Monterrosa et al. 2012 | Chicken prevents anemia | Self-developed | 425 | 0.001 | 1.30 |
| Monterrosa et al. 2012 | Broths nourish my baby | Self-developed | 425 | 0.002 | -0.91 |
| Monterrosa et al. 2012 | Fish prevents anemia | Self-developed | 425 | 0.001 | 1.69 |
| Monterrosa et al. 2012 | After 6 months, breastmilk nourishes my baby | Self-developed | 425 | 0.001 | 1.42 |
| Monterrosa et al. 2012 | If child rejects vegetables, do not feed them again | Self-developed | 425 | 0.23 | -0.20 |
| Rahman et al. 2008 | Child development knowledge | Infant Development Questionnaire | 309 | 0.001 | 1.69 |
| Scheinmann et al. 2009 | A sign that the baby was ready to eat solids | Adapted from Bright Futures Nutrition Series | 272 | 0.05 | -0.45 |
| Scheinmann et al. 2009 | That solids don´t replace formula/breastmilk | Adapted from Bright Futures Nutrition Series | 272 | 0.001 | 1.20 |
| Scheinmann et al. 2009 | When solids become baby´s main foods | Adapted from Bright Futures Nutrition Series | 272 | 0.54 | -0.12 |
| Scheinmann et al. 2009 | The size of a baby´s stomach | Adapted from Bright Futures Nutrition Series | 272 | 0.001 | 0.96 |
| Scheinmann et al. 2009 | That breastfeeding has all the nutrients a baby needs | Adapted from Bright Futures Nutrition Series | 272 | 0.25 | 1.70 |
| Scheinmann et al. 2009 | That breastmilk is easier to digest than formula | Adapted from Bright Futures Nutrition Series | 272 | 0.26 | -0.80 |
| Scheinmann et al. 2009 | That peanutbutter should not be given to a 12 months old | Adapted from Bright Futures Nutrition Series | 272 | 0.06 | 0.27 |
| Scheinmann et al. 2009 | That it would be ok to limit how much a child eats if he/she´s overweight | Adapted from Bright Futures Nutrition Series | 272 | 0.25 | -0.45 |
| Scheinmann et al. 2009 | What food most likely to make baby choke | Adapted from Bright Futures Nutrition Series | 272 | 0.99 | 0.20 |
| Singh et al. 2017 | Know baby should be given only breast milk for 6 months | Self-developed | 1890 | 0.35 | 0.54 |
| Singh et al. 2017 | Know animal-source foods are good for children aged 6-23.9 months | Self-developed | 1890 | 0.96 | 0.26 |
| Singh et al. 2017 | Know fruits and vegetables are good for children aged 6-23.9 months | Self-developed | 1890 | 0.03 | 1.15 |
| Wang Hsiu-Hung et al. 2012 | Childbearing Knowledge Scale | Childbearing Knowledge Scale | 99 | 0.05 | 0.40 |

Table 5: Albatross plot - Diabetes knowledge

| Author, year of publication | Outcomes | Measurement tool | Sample size | p-value | Effect direction |
| --- | --- | --- | --- | --- | --- |
| Frosch et al. 2011 | Diabetes knowledge | Diabetes Knowledge Test (University of Michigan) | 200 | 0.78 | 0.10 |
| Garcia et al. 2015 | Diabetes knowledge | Spoken Knowledge in Low Literacy in Diabetes Scale | 72 | 0.02 | 0.75 |
| Gerber et al. 2005 | Diabetes knowledge - Lower literacy group | Self-developed | 135 | 0.58 | -0.10 |
| Gerber et al. 2005 | Diabetes knowledge - Higher literacy group | Self-developed | 109 | 0.90 | 0.02 |
| Heisler et al. 2014 | Knowledge and beliefs about anti-hyperglycemic medications | Self-developed | 176 | 0.84 | 0.08 |

Table 6: Albatross plot - Food knowledge

| Author, year of publication | Outcomes | Measurement tool | Sample size | p-value | Effect direction |
| --- | --- | --- | --- | --- | --- |
| Au et al. 2017 | Amount of sodium adults should consume daily | Self-developed | 327 | 0.009 | -0.33 |
| Au et al. 2017 | Main source of dietary salt | Self-developed | 327 | 0.007 | 0.25 |
| Backman et al. 2011 | Correct number of cups regarding vegetable and fruit consumption | Self-developed | 385 | 0.01 | 0.30 |
| Backman et al. 2011 | Knowledge: The number of cups equivalent to a handful of fruits and vegetables: one half cup | Self-developed | 385 | 0.01 | 0.40 |
| Backman et al. 2011 | 3 factors determining the amount of fruit and vegetables recommended for individual daily consumption: age, gender, physical activity | Self-developed | 385 | 0.001 | 0.96 |
| Berry et al. 2011 | Nutrition knowledge | Self-developed | 56 | 0.008 | 0.74 |
| Bessems et al. 2020 | Do you know what you can do to eat healthier? (yes/no) | Self-developed | 152 | 0.432 | 0.21 |
| Bessems et al. 2020 | Do you know how to save money on groceries? | Self-developed | 152 | 0.07 | 0.45 |
| Gittelsohn et al. 2010 | Food knowledge | Customer Impact Questionnaire | 84 | 0.21 | 0.30 |
| Havas et al. 2003 | Knowledge fat | Food Frequency Questionnaire | 1104 | 0.001 | 0.28 |
| Havas et al. 2003 | Knowledge fruit and vegetable | Food Frequency Questionnaire | 1104 | 0.009 | 0.12 |

| Havas et al. 2003 | Knowledge fiber | Food Frequency Questionnaire | 1104 | 0.001 | 0.32 |
| --- | --- | --- | --- | --- | --- |
| Howard-Pitney et al. 1997 | Nutrition knowledge | Self-developed | 351 | 0.01 | 1.90 |
| Hughes et al. 2016 | Less vegetables eaten the longer Koreans in USA | Self-developed | 71 | 0.04 | 1.23 |
| Hughes et al. 2016 | Most Fruits and vegetables have almost no sodium | Self-developed | 71 | 0.001 | 4.43 |
| Hughes et al. 2016 | Recommended daily intake for fruits and vegetables varies by physical activity level | Self-developed | 71 | 0.001 | 3.60 |
| Hughes et al. 2016 | Recommended daily intake fruits | Self-developed | 71 | 0.001 | 1 |
| Hughes et al. 2016 | Recommended daily intake vegetables | Self-developed | 71 | 0.001 | 1.45 |
| Kjøllesdal et al. 2010 | Emphasis salad | Self-developed | 198 | 0.001 | 0.70 |
| Kjøllesdal et al. 2010 | Emphasis meat | Self-developed | 198 | 0.008 | 0.823 |
| Kjøllesdal et al. 2010 | Limitation sugar | Self-developed | 198 | 0.53 | 0.15 |
| Kjøllesdal et al. 2010 | Limitation white flour | Self-developed | 198 | 0.001 | 0.74 |
| Kjøllesdal et al. 2010 | Limitation white rice | Self-developed | 198 | 0.054 | 0.30 |
| Nguyen et al. 2018 | Husbands food knowledge, total score | Self-developed | 1307 | 0.001 | 0.97 |
| Ratnapradipa et al. 2011 | Food handling knowledge, total score | Self-developed | 32 | 0.001 | 0.30 |
| Tessaro et al. 2007 | Number of fruit and vegetable servings per day | Validated tool from NHANES data^2^ | 262 | 0.32 | 0.08 |
| Tessaro et al. 2007 | Fat grams intake | Validated tool from NHANES data^2^ | 262 | 0.33 | 0.16 |
| Tessaro et al. 2007 | Knowledge about dietary fat | Validated tool from NHANES data^2^ | 262 | 0.008 | 0.20 |

| Tessaro et al. 2007 | Knowledge regarding nutrition in HIV patients | Validated tool from NHANES data^2^ | 262 | 0.001 | 1.02 |
| --- | --- | --- | --- | --- | --- |
| Tessaro et al. 2007 | Knowledge of low fat and fruit and vegetable intake | Validated tool from NHANES data^2^ | 262 | 0.70 | 0.04 |

Table 7: Albatross plot - HIV/HPV knowledge

| Author, year of publication | Outcomes | Measurement tool | Sample size | p-value | Effect direction |
| --- | --- | --- | --- | --- | --- |
| Bahromov et al. 2011 | HIV/AIDS knowledge | Self-developed (adapted from previous surveys) | 60 | 0.18 | 0.46 |
| Bahromov et al. 2011 | Condom use knowledge | Self-developed (adapted from previous surveys) | 60 | 0.001 | 1.44 |
| Bogale et al. 2011 | HIV/AIDS knowledge | Self-developed true/false questionnaire | 417 | 0.001 | 0.30 |
| Carey et al. 2000 | HIV knowledge | HIV knowledge questionnaire | 77 | 0.012 | 0.59 |
| Choi et al. 2013 | Knowledge of cervical cancer /HPV knowledge | Tool developed by Kim and Ahn (2007)^3^ | 56 | 0.001 | 0.74 |
| Cianelli et al. 2012 | HIV knowledge | Scale created by Heckman et al.^4^ | 400 | 0.001 | 0.78 |
| Dancy et al. 2000 | Condom use knowledge | Self-developed | 196 | 0.021 | 0.25 |
| Flaskerud et al. 1990 | Total (AIDS) knowledge | Self-developed | 712 | 0.001 | 0.45 |
| Lawrence et al. 2001 | AIDS knowledge Theory of Gender and Power (TGP) vs control | Self-developed | 297 | 0.97 | 0 |
| Lawrence et al. 2001 | AIDS knowledge Social Learning Theory (SLT) vs control | Self-developed | 297 | 0.001 | 0.38 |
| Lawrence et al. 2001 | AIDS knowledge Cognitive Behavioral Modification (CBM) vs control | Self-developed | 297 | 0.02 | 0.26 |
| Li & Lin et al. 2014 | HIV knowledge | HIV knowledge questionnaire | 641 | 0.001 | 0.57 |
| Li & Li et al. 2014 | HIV-related knowledge | KAB on HIV/AIDS for migrants | 529 | 0.05 | 4.28 |
| Nyamathi et al. 1993 | Perfect (AIDS) knowledge | Self-developed (adapted from previous studies) | 858 | 0.001 | -0.45 |
| Nyamathi et al. 1999 | Perfect (AIDS) knowledge | Self-developed (adapted from previous studies) | 410 | 0.001 | -0.46 |
| Pokharel et al. 2019 | HIV knowledge | Self-developed | 66 | 0.02 | 0.58 |
| Sanderson et al. 2015 | HPV knowledge | Self-developed | 373 | 0.14 | 0.05 |
| Van Servellen et al. 2003 | Level of health literacy - HIV knowledge/misconceptions | Self-developed true/false instrument | 81 | 0.01 | 0.61 |
| Van servellen et al. 2005 | Global HIV disease/treatment knowledge | Self-developed | 69 | 0.2 | -0.29 |
| Van servellen et al. 2005 | HIV treatment-related knowledge subscale | Modified REALM | 69 | 0.3 | 0.24 |
| Van servellen et al. 2005 | Recognition HIV terms | Modified REALM | 69 | 0.8 | 0.05 |
| Van servellen et al. 2005 | Understanding HIV terms | Modified REALM | 69 | 0.96 | 0.01 |
| Van servellen et al. 2005 | Knowledge risk getting sicker | Modified REALM | 69 | 0.12 | -0.38 |
| Zhang et al. 2013 | HIV/AIDS knowledge (Score) 9-11 Traditional VCT vs community mobilization plus comprehensive VCT | Self-developed | 1273 | 0.001 | 1.36 |

KAB: Knowledge, attitudes and sexual behavior

# References

^1^ Evaluation of a fotonovela to increase depression knowledge and reduce stigma among hispanic adults." Journal of Immigrant and Minority Health 15(2): 398-406.

^2^ Block G, Hartman A, Dresser C, et al. A data-based approach to diet questionnaire design and testing. Am J Epidemiol. 1986; 124:453-469

^3^ Kim HW, Ahn HY (2007). Study on the knowledge of human papilloma virus in female university

^4^Heckman T, Sikkema K, Kelly J, Fuqua R, Mercer M, Hoffmann R, et al. Predictors of condom use and human immunodeficiency virus test seeking among women living in inner-city public housing developments. Sexually Transmitted Diseases. 1996; 23(5):357–365. [PubMed: 8885065]
