# Supplementary material for: Interventions to promote health literacy among working-age populations experiencing socioeconomic disadvantage: systematic review
Source: Front Public Health. 2024 Feb 19;12:1332720. doi: 10.3389/fpubh.2024.1332720 (PMC10909862; doi:10.3389/fpubh.2024.1332720)
Supplement: Supplementary file 1 [file Data_Sheet_1.ZIP › Supplementary file 5_Albatross_plots_by RoB.docx]

# Supplementary file 5 – Analysis of studies according to risk of bias

# Interventions to promote health literacy among socioeconomically disadvantaged working-age population groups: Systematic review

Himal Singh^1*^, Florence Samkange-Zeeb^2^, Jonathan Kolschen^1^, Ruben Herrmann^1^, Wiebke Hübner^2^, Núria, Pedrós Barnils^1^, Tilman Brand^2^, Hajo Zeeb^2,3^, Benjamin Schüz^1,3^

^1^Institute of Public Health and Nursing Research, University of Bremen, Bremen, Germany

^2^Department of Prevention and Evaluation, Leibniz Institute for Prevention Research and Epidemiology – BIPS, Bremen, Germany

^3^Health Sciences Bremen, University of Bremen, Bremen, Germany

For a large part of the included studies, we assessed a high risk of bias. In the following analysis we reran the albatross plots and categorized the studies by their risk of bias.

Notably, studies with a low or moderate risk of bias often showed smaller effect sizes compared to studies where we assessed a higher risk of bias.

This analysis underlines the importance of interpreting the results cautiously and especially underlines the need of high-quality studies needed to identify effective interventions strategies to promote health literacy among socioeconomically disadvantaged populations.

## Functional health literacy


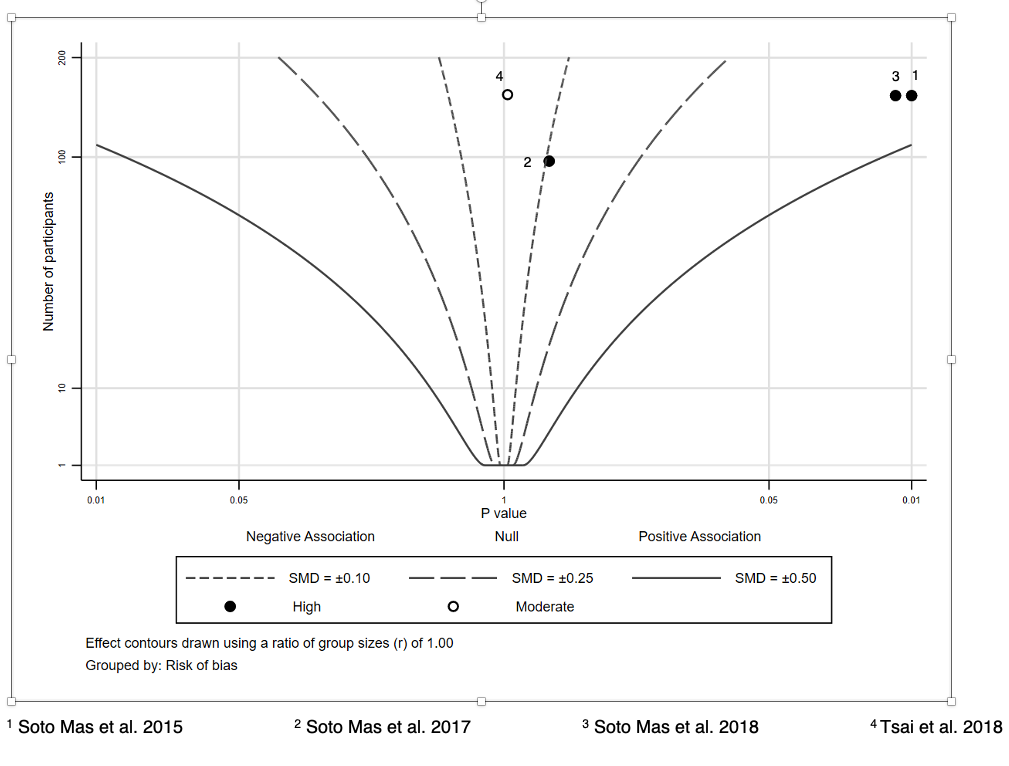


Figure 1:Albatross-plot -Functional health literacy

## Mental health literacy


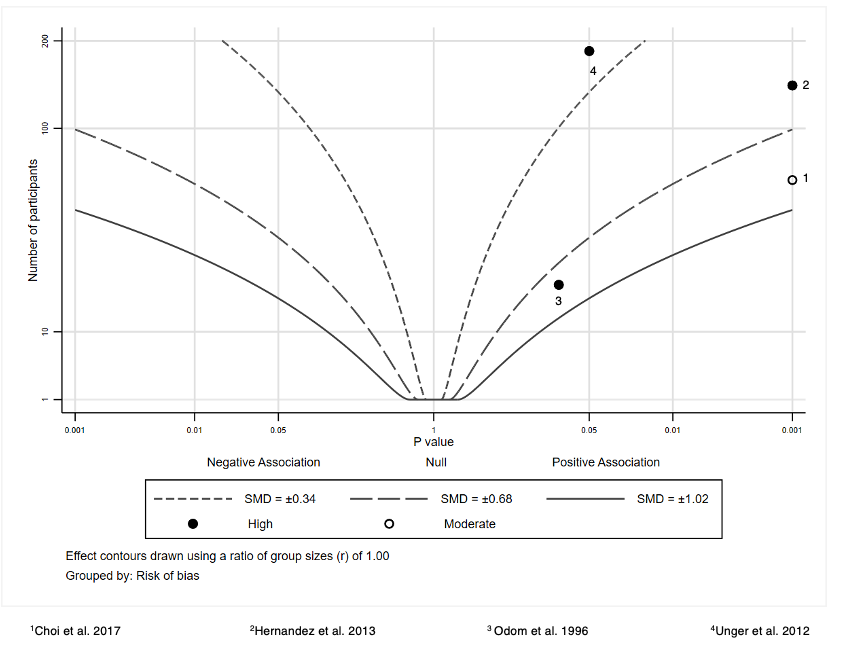


Figure 2: Albatross plot - Mental health literacy

## Cancer screening knowledge


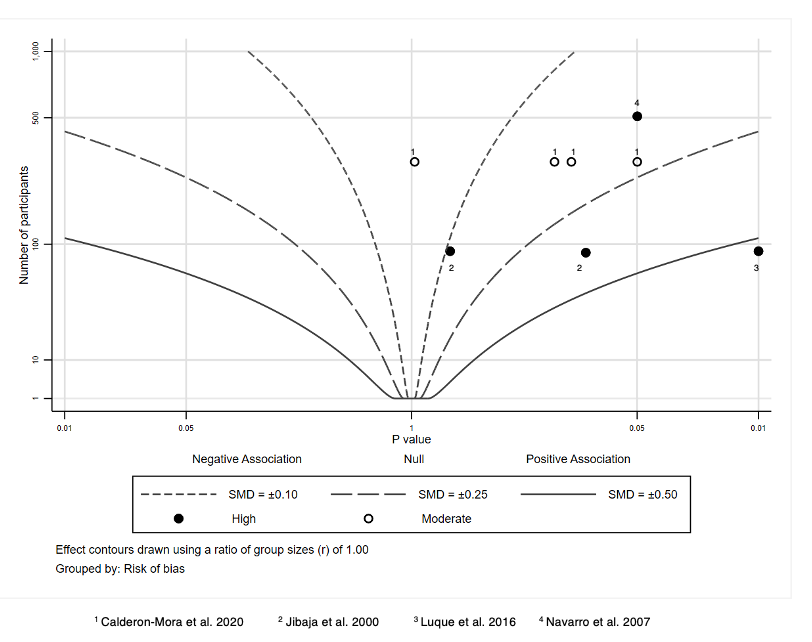


Figure 3: Albatross plot - Cancer screening knowledge

## Childfeeding knowledge


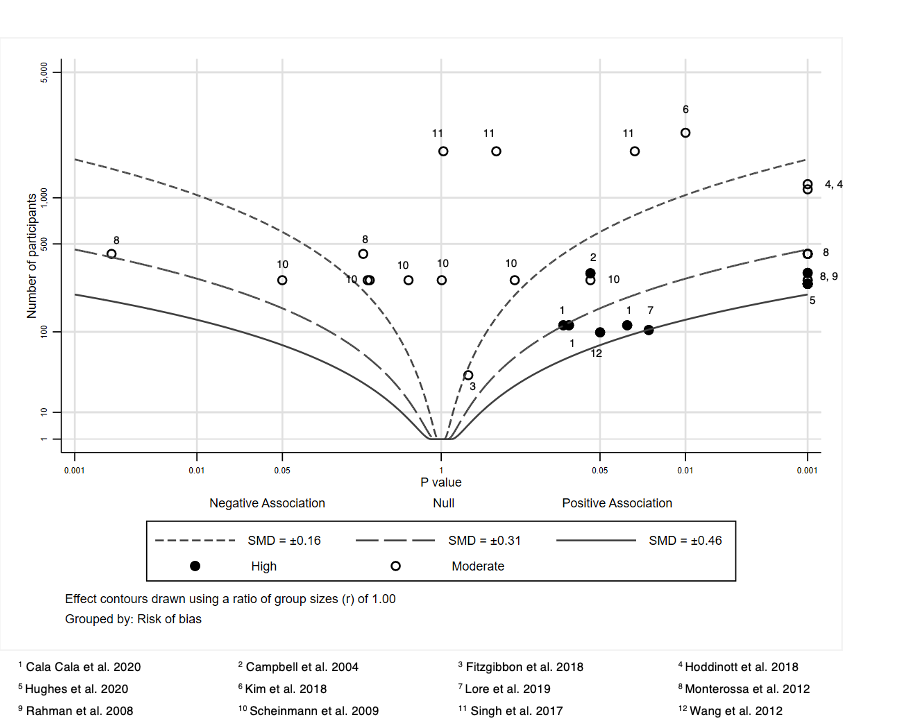


Figure 4: Albatross plot - Childfeeding knowledge

## Diabetes knowledge


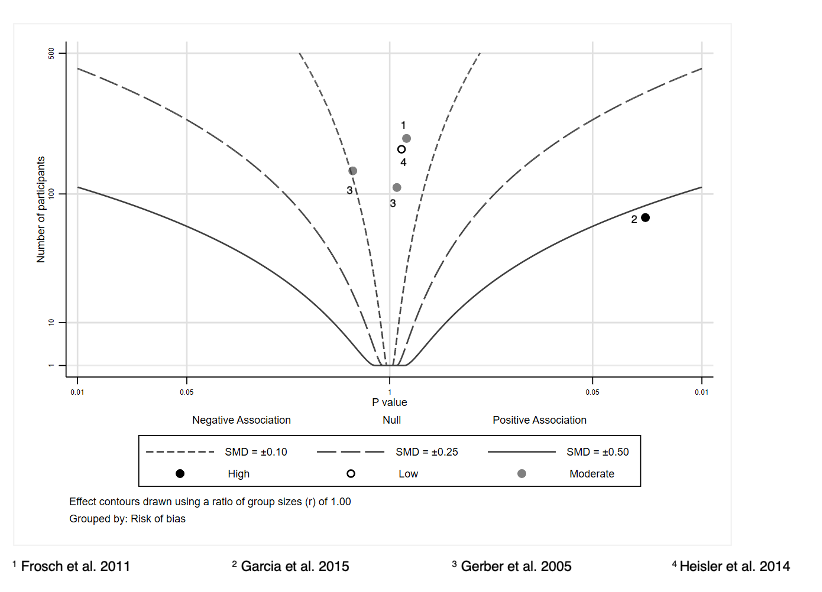


Figure 5: Albatross plot – Diabetes knowledge

## Food knowledge


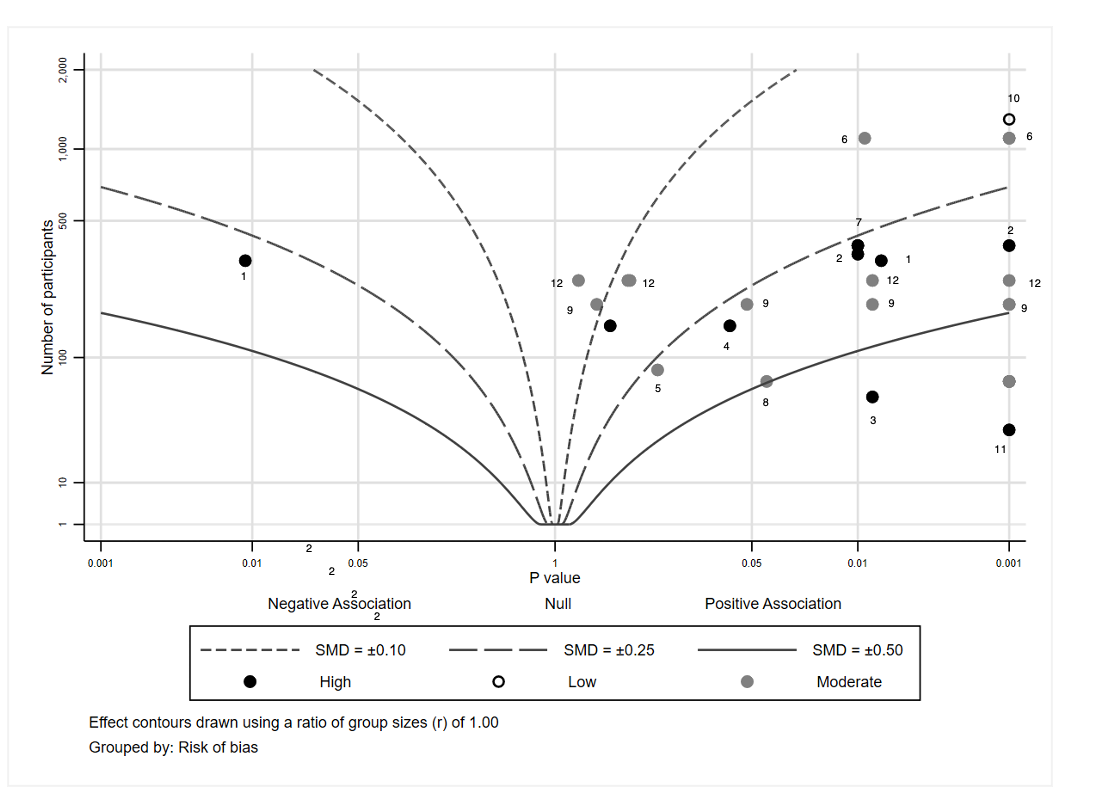


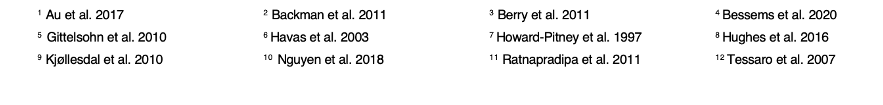


Figure 6: Albatross plot – Food knowledge

## HIV knowledge


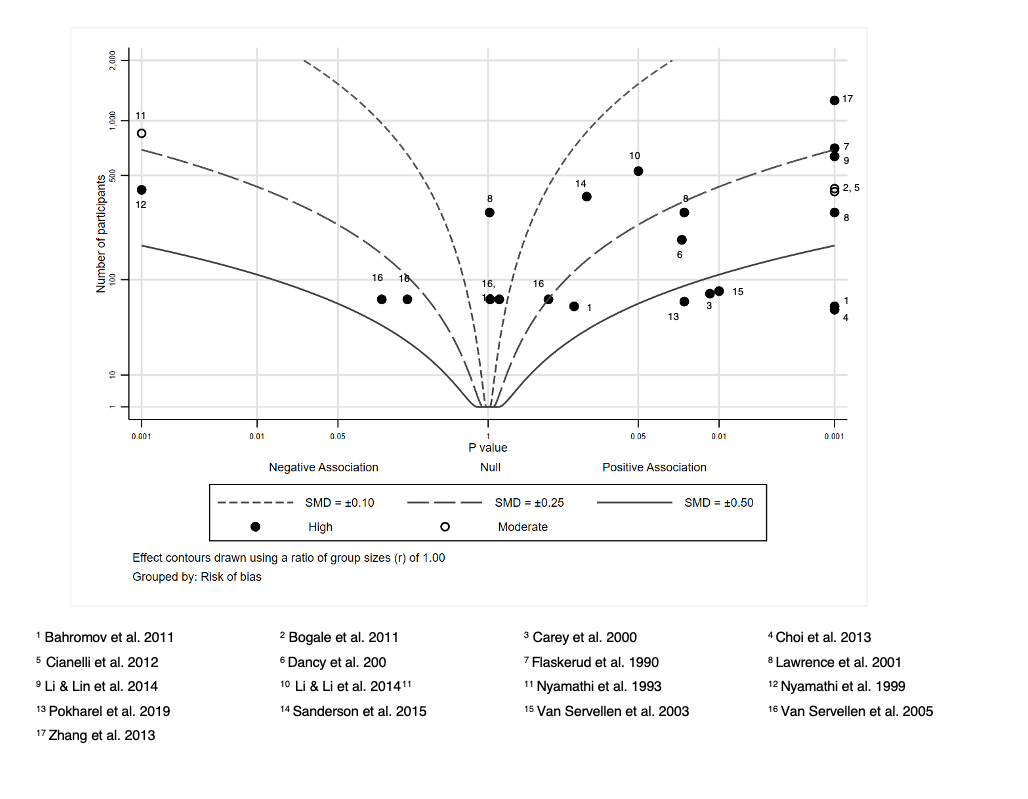


Figure 7 - Albatross plot – HIV knowledge
