# Supplementary material for: Interventions to promote health literacy among working-age populations experiencing socioeconomic disadvantage: systematic review
Source: Front Public Health. 2024 Feb 19;12:1332720. doi: 10.3389/fpubh.2024.1332720 (PMC10909862; doi:10.3389/fpubh.2024.1332720)
Supplement: Supplementary file 1 [file Data_Sheet_1.ZIP › Supplementary file 6_Sensitivity analysis.docx]

# Supplementary file 6 – Sensitivity analysis

# Interventions to promote health literacy among socioeconomically disadvantaged working-age population groups: Systematic review

Himal Singh^1*^, Florence Samkange-Zeeb^2^, Jonathan Kolschen^1^, Ruben Herrmann^1^, Wiebke Hübner^2^, Núria, Pedrós Barnils^1^, Tilman Brand^2^, Hajo Zeeb^2,3^, Benjamin Schüz^1,3^

^1^Institute of Public Health and Nursing Research, University of Bremen, Bremen, Germany

^2^Department of Prevention and Evaluation, Leibniz Institute for Prevention Research and Epidemiology – BIPS, Bremen, Germany

^3^Health Sciences Bremen, University of Bremen, Bremen, Germany

## Selection of studies

11 articles excluded during the full-text screening did not report the mean age of the population but only age groups or the age range which was not between 18 and 65 years (1-11). As we could not clearly identify if the study population matched our inclusion criteria, we looked at these articles separately and compared the intervention strategies used to promote health literacy and health knowledge outcomes to our main findings.

## Study characteristics

Similar as in the main analyses most of the studies considered for the analysis were conducted in the USA (n=4) (5, 7, 8, 10). Further studies were conducted in Australia (2), Bangladesh (3), China (1), Kenya (11), Nigeria (9), Netherlands (4) and Turkey (6).

7 of the 11 studies had a randomized controlled study design with the remainder using a non-randomized controlled design. Health outcomes addressed were heterogeneous including AIDS knowledge, breast cancer knowledge, child feeding and maternal nutrition knowledge, diabetes knowledge, food knowledge, hepatitis- knowledge, hypertension knowledge, malaria knowledge, oral health knowledge, parasitic diseases knowledge and smoking-related knowledge,

## Effective interventions

We summarized 16 health knowledge outcomes from 10 studies (Figure 1).

Statistically significant improvements were shown in seven outcomes from seven studies (1, 5-7, 9-11).

Large effects were shown while improving breast cancer knowledge, parasite knowledge and malaria knowledge (5, 6, 9). Implemented interventions included a breast educational program based on Bandura´s theory of self-efficacy and Freire’s empowerment pedagogy conducted by bilingual gatekeepers, (5), an educational program implemented via home visits with by the researchers to promote parasitic diseases knowledge (6) and a malaria educational program consisting of lectures, role-plays, interactive sessions, and demonstrations and implemented by community volunteers (9).

Effective intervention strategies were similar as in the main findings, enabling easy access to educational sessions and materials (via home visits e.g. (6) ) and overcoming language barriers via bilingual gatekeepers. (5).

Also, all interventions with a statistically significant health knowledge improvement included a face-to-face component. These results are in-line with the main findings.


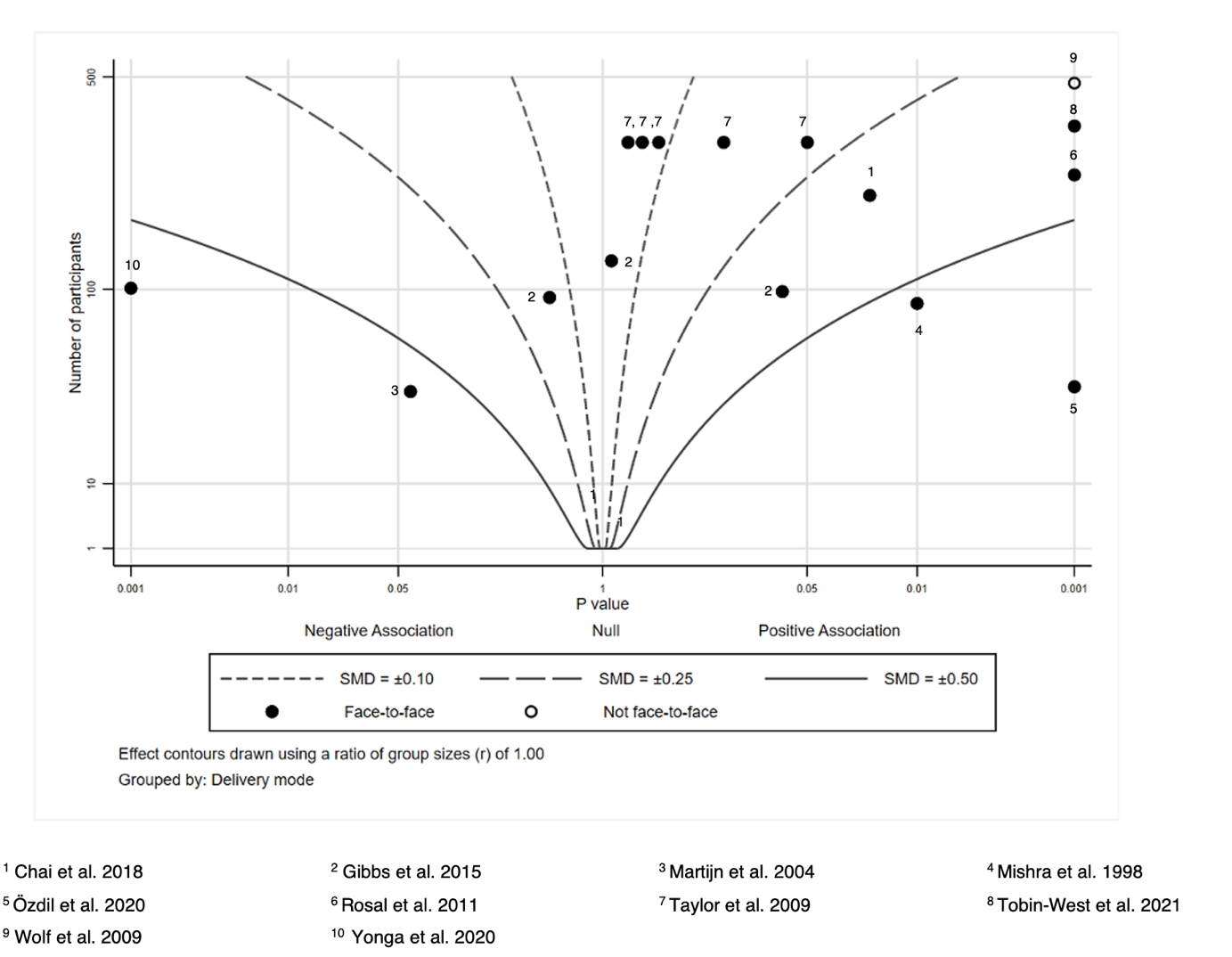


Figure 1: Albatross plot - Sensitivity analysis

# References

1. Chai W, Zou G, Shi J, Chen W, Gong X, Wei X, et al. Evaluation of the effectiveness of a WHO-5A model based comprehensive tobacco control program among migrant workers in Guangdong, China: A pilot study. BMC Public Health. 2018;18(1).

2. Gibbs L, Waters E, Christian B, Gold L, Young D, de Silva A, et al. Teeth Tales: a community-based child oral health promotion trial with migrant families in Australia. BMJ Open. 2015;5(6):e007321.

3. Hoddinott J, Ahmed A, Roy S. Randomized control trials demonstrate that nutrition-sensitive social protection interventions increase the use of multiple-micronutrient powders and iron supplements in rural pre-school Bangladeshi children. Public Health Nutrition. 2018;21(9):1753-61.

4. Martijn C, De Vries NK, Voorham T, Br, sma J, Meis M, et al. The effects of AIDS prevention programs by lay health advisors for migrants in the Netherlands. Patient Education and Counseling. 2004;53(2):157-65.

5. Mishra SI, Chavez LR, Magaña JR, Nava P, Burciaga Valdez R, Hubbell FA. Improving breast cancer control among Latinas: evaluation of a theory-based educational program. Health Educ Behav. 1998;25(5):653-70.

6. Özdil K, Karataş N, Zincir H. Low socioeconomic level and enterobius vermicularis: A interventional study to children and their mothers in home. Zoonoses and Public Health. 2020;67(8):882-91.

7. Rosal MC, Ockene IS, Restrepo A, White MJ, Borg A, Olendzki B, et al. Randomized trial of a literacy-sensitive, culturally tailored diabetes self-management intervention for low-income Latinos: Latinos en control. Diabetes Care. 2011;34(4):838-44.

8. Taylor VM, Hislop TG, Tu S-P, Teh C, Acorda E, Yip M-P, et al. Evaluation of a Hepatitis B Lay Health Worker Intervention for Chinese Americans and Canadians. Journal of Community Health. 2009;34(3):165-72.

9. Tobin-West CI, Briggs N. Effectiveness of trained community volunteers in improving knowledge and management of childhood malaria in a rural area of Rivers State, Nigeria. Niger J Clin Pract. 2021;18(5):651-8.

10. Wolf RL, Lepore SJ, ergrift JL, Basch CE, Yaroch AL, Wolf R, et al. Tailored telephone education to promote awareness and adoption of fruit and vegetable recommendations among urban and mostly immigrant black men: a randomized controlled trial. Preventive Medicine. 2009;48(1):32-8.

11. Yonga G, Okello FO, Herr JL, Mulvaney A, Ogola EN. Healthy heart Africa: A prospective evaluation of programme outcomes on individuals' hypertension awareness, screening, diagnosis and treatment in rural Kenya at 12 months. Cardiovascular Journal of Africa. 2020;31(1):9-15.
